# Attenuation of Nrf2/Keap1/ARE in Alzheimer’s Disease by Plant Secondary Metabolites: A Mechanistic Review

**DOI:** 10.3390/molecules25214926

**Published:** 2020-10-24

**Authors:** Sajad Fakhri, Mirko Pesce, Antonia Patruno, Seyed Zachariah Moradi, Amin Iranpanah, Mohammad Hosein Farzaei, Eduardo Sobarzo-Sánchez

**Affiliations:** 1 Pharmaceutical Sciences Research Center, Health Institute, Kermanshah University of Medical Sciences, Kermanshah 6734667149, Iran; sajad.fakhri@kums.ac.ir (S.F.); zmoradi@kums.ac.ir (S.Z.M.); 2 Department of Medicine and Aging Sciences, University G. d’Annunzio CH-PE, 66100 Chieti, Italy; mirko.pesce@unich.it; 3 Medical Biology Research Center, Health Technology Institute, Kermanshah University of Medical Sciences, Kermanshah 6734667149, Iran; 4 Student Research Committee, Kermanshah University of Medical Sciences, Kermanshah 6714415153, Iran; amin.iranpanah75@gmail.com; 5 Laboratory of Pharmaceutical Chemistry, Department of Organic Chemistry, Faculty of Pharmacy, University of Santiago de Compostela, 15782 Santiago de Compostela, Spain; eduardo.sobarzo@ucentral.cl; 6 Instituto de Investigación e Innovación en Salud, Facultad de Ciencias de la Salud, Universidad Central de Chile, Santiago 8330507, Chile

**Keywords:** Alzheimer’s disease, oxidative stress, Nrf2, Keap1, antioxidant response elements, phytochemicals, secondary metabolites, pharmacology

## Abstract

Alzheimer’s disease (AD) is a progressive neuronal/cognitional dysfunction, leading to disability and death. Despite advances in revealing the pathophysiological mechanisms behind AD, no effective treatment has yet been provided. It urges the need for finding novel multi-target agents in combating the complex dysregulated mechanisms in AD. Amongst the dysregulated pathophysiological pathways in AD, oxidative stress seems to play a critical role in the pathogenesis progression of AD, with a dominant role of nuclear factor erythroid 2-related factor 2 (Nrf2)/Kelch-like ECH-associated protein-1 (Keap1)/antioxidant responsive elements (ARE) pathway. In the present study, a comprehensive review was conducted using the existing electronic databases, including PubMed, Medline, Web of Science, and Scopus, as well as related articles in the field. Nrf2/Keap1/ARE has shown to be the upstream orchestrate of oxidative pathways, which also ameliorates various inflammatory and apoptotic pathways. So, developing multi-target agents with higher efficacy and lower side effects could pave the road in the prevention/management of AD. The plant kingdom is now a great source of natural secondary metabolites in targeting Nrf2/Keap1/ARE. Among natural entities, phenolic compounds, alkaloids, terpene/terpenoids, carotenoids, sulfur-compounds, as well as some other miscellaneous plant-derived compounds have shown promising future accordingly. Prevailing evidence has shown that activating Nrf2/ARE and downstream antioxidant enzymes, as well as inhibiting Keap1 could play hopeful roles in overcoming AD. The current review highlights the neuroprotective effects of plant secondary metabolites through targeting Nrf2/Keap1/ARE and downstream interconnected mediators in combating AD.

## 1. Introduction

As a progressive dysfunction in neurons and cognition, Alzheimer’s disease (AD) is a leading cause of death and disability [1]. Inflammation, apoptosis, and oxidative stress are major dysregulated pathways involved in the progression of AD, which the latter is linked to several neurodegenerative diseases and seems to play key destructive roles [2]. Consequently, the activation of endogenous antioxidant mediators is a critical strategy in combating related complications [3]. In this regard, the inflammatory and oxidative hypothesis in AD seem to be of great importance. The oxidative pathways mainly pass through the byproducts of the electron transport chain, including hydrogen peroxide radicals (H_2_O_2_), superoxide radical (O_2_^−^.) and the hydroxyl radicals (OH·) [4]. These pathways/mediators seem to be regulated by nuclear factor erythroid 2-related factor 2 (Nrf2) as an upstream mediator. Nrf2, in turn, is tightly regulated by Kelch-like ECH-associated protein-1 (Keap1) and antioxidant responsive elements (ARE) [5,6,7]. Under oxidative stress, Keap1 modification causes conformational changes to disrupt the Keap1/Nrf2 interaction and prevents Nrf2 degradation. This leads to nuclear translocation of Nrf2, binding to ARE, followed by the activation of antioxidant enzymes. Besides, increased level of oxidative stress also elevates the production of inflammatory mediators, including interleukin (IL)-1β, IL-6, as well as tumor necrosis factor-α (TNF-α) and subsequently, contribution of phosphatidylinositide 3-kinases (PI3K)/Akt/mammalian target of rapamycin (mTOR). The resulted inflammation induced by pro-inflammatory cytokines, in turn, causes synaptic damage and neuronal loss toward the progression of AD [8]. Oxidative stress could also trigger apoptotic pathways (e.g., Bax, Bad, and caspase) and amyloid β peptides (Aβ_1–42_), as well as tau phosphorylation toward neuronal death. Overall, oxidative stress could be considered an upstream regulator of dysregulated destructive pathways in AD, and Nrf2/ARE pathway as a major inhibitory orchestrate against AD. In addition to the inflammatory and oxidative hypothesis in AD, the tau hypothesis, amyloid-cascade and cholinergic hypothesis seem to play more critical roles in the pathogenesis of AD. The tau hypothesis declares that abnormal or excessive phosphorylation of tau leads to the transformation of normal adult tau into paired helical filament (PHF)-tau and intracellular neurofibrillary tangles (NFT). This procedure facilitates the interaction of tau isomers with tubulin to stabilize microtubule assembly, and leading to cell death/dementia. As mentioned, the amyloid-cascade, as the process of forming Aβ plaques peptide aggregation resulted from proteolytic cleavages of Aβ precursor protein (APP), is also central to AD pathology. Additionally, dysregulation in the function of basal forebrain cholinergic cell (cholinergic hypothesis), as well as other neurotransmitters (e.g., glutamate, norepinephrine, dopamine) are other pivotal theories leading to AD [9].

Prevailing studies are showing the pivotal role of oxidative stress in triggering other causative destructive pathways. So targeting oxidative stress and its major pathway (Nrf2/Keap1/ARE) is of great importance. Despite advances in clinical studies, no effective treatment has yet been provided. Considering the complexity of destructive oxidative pathways behind AD, single-target antioxidants seemed not to be appropriate therapeutics in AD. Prevailing evidence has been established toward effective AD treatment among natural sources to activate Nrf2 and ARE pathway. During recent decades, the plant kingdom has been a major source of active metabolites in targeting oxidative stress in neurodegenerative diseases. Phytochemicals are structurally diverse compounds with potential pharmacological mechanisms and beneficial effects on human health. These compounds are of potential importance in drug discovery because they provide a large range of compounds with therapeutic potentials for the treatment of diverse diseases [10]. They have also attracted particular attention in the management/control of AD by targeting oxidative stress mediators [11]. Several classes of plant-derived secondary metabolites, including phenolic compounds, alkaloids, terpene/terpenoids, carotenoids, sulfur-compounds, and other miscellaneous compounds have successfully targeted the Nrf2/Keap1/ARE pathway and related interconnected mediators in AD.

Previous studies have introduced Nrf2 as a therapeutic target in chronic diseases [12,13] and AD [14,15,16,17], with no focus on the role of natural secondary metabolites. The present review reveals the role of Nrf2/Keap1/ARE and interconnected pathways as therapeutic targets in the treatment of AD. A promising perspective for plant secondary metabolites has also been provided to find potential therapeutic agents in the treatment of AD.

## 2. Study Design

A comprehensive review was performed, using electronic databases, including Scopus, PubMed, Medline, Web of Science, and related articles regarding Alzheimer’s disease, as well as the phytochemicals effects. The keywords (“Alzheimer’s disease” OR “Nrf2” OR “Keap1”, OR “ARE”) [title/abstract/keywords] were used. All the interconnected mediators to Nrf2/Keap1/ARE as well as all the phytochemicals, including phenolic compounds, alkaloids, terpene/terpenoids, carotenoids, sulfur-compounds were found in the whole text. Overall, the inclusion criteria were phytochemicals affecting Nrf2/Keap1/ARE and cross-talk pathways, as major involved pathways of Alzheimer’s disease in all the study types, including in vitro, in vivo, and clinical trials. Data were collected without date and language restrictions until August 2020. The reference lists and citation hand search with the authors’ expertise in signaling pathways and Nrf2/Keap1/ARE as pivotal therapeutic targets of phytochemicals in Alzheimer’s disease were also used to complete the search on the electronic databases.

## 3. Nrf2/Keap1/ARE Pathway and Interconnected Mediators in AD

Oxidative stress seems to be an upstream orchestrate of neurodegeneration either by activating the inflammatory and apoptotic pathways in neurodegenerative diseases. Prevailing studies have shown the critical role of overactivated reactive oxygen species (ROS)/reactive nitrogen species (RNS) in causing cell death, during pathological conditions. Consequently, the oxidative phosphorylation of mitochondria, as well as parallel dysregulated pathways, could be major sources of produced ROS/RNS. Of those parallel hallmarks of AD, amyloid-beta (Aβ) plays a major role in activating oxidative mediators [18]. On the other hand, Nrf2 is now identified as a key upstream defence mediator against oxidative pathways. Essentially, Nrf2 is a member of the cap’n’collar (CNC) family of basic region-leucine zipper transcription factors, responsible for activating the antioxidant enzymes, including NAD(P)H quinone oxidoreductase-1 (NQO1), superoxide dismutase-1 (SOD1), heme oxygenase-1 (HO-1), glutathione *S*-transferase (GST), catalase (CAT), and glutathione peroxidase (GPx), as downstream antioxidant enzymes [19,20]. In normal condition, Keap1 is a repressor protein that binds to an E3 ubiquitin ligase complex (Rbx-1) via cullin-3 to promote the degradation of Nrf2. However, in the situation of oxidative stress, Keap1 undergoes conformational changes toward the release of Nrf2 and subsequent activation of ARE. Several interconnected mediators could activate Nrf2 through phosphorylation and proceed antioxidative responses. Amongst those mediators, PI3K [21], c-Jun N-terminal kinase (JNK), extracellular regulated kinase (ERK) and mitogen-activated protein kinases (MAPKs) [22] are key kinases that phosphorylate Nrf2 and allowing to its nucleus transportation. On the other hand, some kinases are overactivated during the pathological conditions to degrade Nrf2 via Keap1-dependent and independent manners. For instance, glycogen synthase kinase 3-beta (GSK-3β) has been shown to make proteasomal degradation of Nrf2 [23], while p38 MAPK stabilizes the Keap1/Nrf2 interaction toward oxidative responses [22]. GSK-3β plays a key role in the advancement/etiology of AD. From the mechanistic point of view, GSK-3β is in a near link with Aβ deposition and tau hyper-phosphorylation, which is associated with AD pathogenesis. Additionally, GSK-3β affects the oxidative stress, as a major hypothesis in AD. In this line, growing studies have paid to build a connection between GSK-3β and Nrf2 in AD pathology. Consistently, GSK-3β suppression was found to increase Nrf2 and some downstream AREs in brain cortex during AD [24]. Nowadays, usefulness of dual GSK-3β inhibitors/Nrf2 inducers are shown in various reports against AD.

From the inflammatory point of view, nuclear factor-κB (NF-κB) has also been shown to co-transported with Keap1 into nuclei regarding trapping Nrf2 [25]. Alternatively, Nrf2, in turn, could suppress inflammatory pathways through activating anti-inflammatory mediators (e.g., IL-10) and inhibiting inflammatory ones (TNF-α, IL-6, IL-1β). Besides, Nrf2 has also shown inhibitory effects on apoptotic mediators (e.g., Bax, Bad) and stimulatory effects on antiapoptotic factors (e.g., Bcl-2) [20]. In general, the Nrf2/ARE pathway is down-regulated within hippocampal neurons during AD [26]. Therefore, inducing Nrf2/ARE could be a valuable strategy for the treatment of AD.

**Figure 1:**
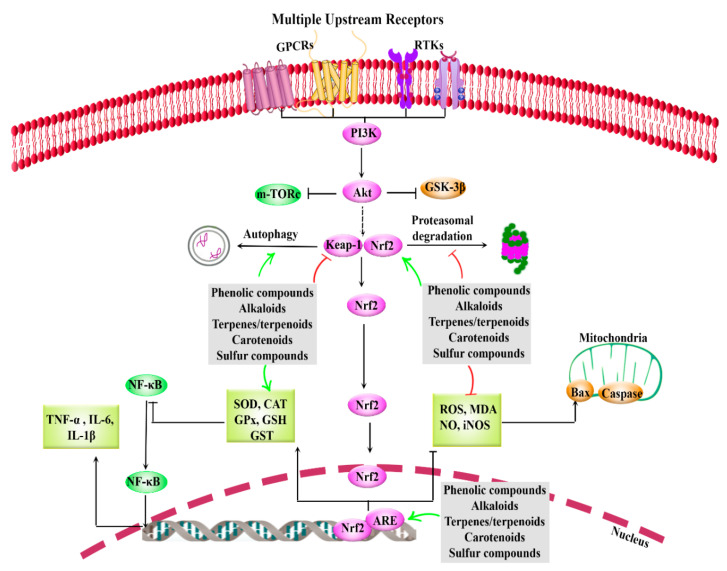
General overview of Nrf2/Keap1/ARE and interconnected pathways, how to be targeted by phytochemicals. Phytochemicals activate Nrf2, ARE (e.g., SOD, CAT, GPx, GSH, GST) and autophagy, while inhibits Keap1, oxidative mediators (e.g., ROS, MDA, NO, iNOS) and inflammation (IL, TNF-α, NF-κB). ↑ green: Activate or up-regulation, ⊥ red: inhibit or down-regulation, ARE: antioxidant response element, CAT: catalase, GPCRs: G protein-coupled receptors, GPx: glutathione peroxidase, GSH: glutathione, GSK-3β: glycogen synthase kinase 3-beta, GST: glutathione *S*-transferase, IL: interleukin, iNOS: inducible nitric oxide synthase, Keap1: Kelch-like ECH-associated protein-1, MDA: malondialdehyde, mTORc: mammalian target of rapamycin, NF-κB: nuclear factor-κB, NO: nitric oxide, Nrf2: nuclear factor erythroid 2-related factor 2, ROS: reactive oxygen species, RTKs: receptor tyrosine kinase, SOD: superoxide dismutase, TNF-α: tumor necrosis factor-α.

Considering the multiple dysregulated pathways interconnected with Nrf2, providing multi-target therapeutic agents is of great importance. Figure 1 indicates the general view of Nrf2/Keap1/ARE and related interconnected mediators in AD.

## 4. Phytochemicals Affecting Nrf2/Keap1/ARE Pathway to Combat AD

Considering the critical role of Nrf2/ARE in preventing the pathogenesis of AD, several classes of phytochemicals have shown a bright future in targeting those mediators, thereby combating AD. Amongst those plant-derived secondary metabolites phenolic compounds, alkaloids, terpenes/terpenoids, carotenoids, and sulfur compounds are of great importance.

### 4.1. Phenolic Compounds

Phenolic compounds have been considered of the greatest group of plant secondary metabolites that are extensively distributed in various parts of the plants. In addition to their undeniable roles in the plant’s defense, they can also contribute flavor, color, and astringency of fruits to show an impressive role in accelerating pollination, antifungal and antibacterial activities [27]. Furthermore, phenolic compounds have shown several biological activities, such as cardioprotective, anti-inflammatory, antioxidant, immune system promoting, and anti-carcinogenic effects [28]. Curcumin, quercetin, resveratrol, naringin, naringenin, and chalcones are some of the key ingredients belonging to this class with hopeful Nrf2/Keap1/ARE-mediated roles in the treatment of neurodegenerative diseases, especially AD [29].

#### 4.1.1. Curcumin

Curcumin is one of the most impressive natural polyphenols acquired from *Curcuma longa* L., that can modify and modulate various pharmacological and biological targets, such as genes and cytokines, growth factors, and transcription factors [27,30]. Curcumin suppresses and adjusts the inflammatory processes via modulating several pro-inflammatory mediators like cyclooxygenase-2 (COX-2), TNF-α, and IL-8 [30]. Additionally, it was documented that curcumin can effectively decrease the aggregation of Aβ and other dysregulated proteins, regarding targeting neurodegenerative disorder, primarily AD [29,31]. Sarkar et al. investigated the advantages of using curcumin in preventing and decreasing the neurotoxicity on the SH-SY5Y and IMR-32 cell lines. Their results emphasized that it can increase the expression of DNA repair enzymes APE1, and poly(ADP-Ribose) polymerase 1 (PARP1), as well as the activation of ARE via up-regulation of Nrf2 [32]. Also, in a similar study, the beneficial effects of curcumin analogues were inquired and results showed that interfering with Nrf2/Keap1/HO-1 signaling pathway is the main mechanism of these analogues to diminish the oxidative stress induced by Aβ_25–35_, in vitro [33]. Besides, curcumin properly protected neuronal differentiated human SK-N-SH cells from ROS. The in vivo advantages showed that it can also reduce the activity of caspase-3 and caspase-7 along with the levels of H_2_O_2_ in the brain. Curcumin also enhanced the concentrations of GSH and the ratio of free to oxidized GSH, in vivo [34]. In a similar study, curcumin decreased the damages caused by oxidative stress, ROS, malondialdehyde (MDA), mitochondrial dysfunction, and increased levels of thiol [35]. Poly(lactic-co-glycolic acid) nanoparticles of curcumin also showed a remarkable amelioration of the recognition and spatial memory in the mice model of AD. Decreasing the IL-6, ROS, Aβ level, TNF-α, and increasing the SOD activities are some of the important mechanisms suggested for the anti-AD effect of curcumin [36]. Moreover, it was documented that curcumin, in combination with berberine, significantly decreased the inflammation and oxidative stress and increased the AMPK signaling in AD-induced mice [37]. Besides, interfering with Nrf2/ARE signaling pathway was reported as the main neuroprotection mechanism of curcumin in an animal’s model of traumatic brain injury [38]. Sahin et al. showed that curcumin attenuated the in vivo heat stress through the Nrf2/HO-1 pathway and interconnected oxidative stress mediators [39]. Di-*O*-demethylcurcumin is another analog of curcumin that showed a neuroprotective potential via the activation of Nrf2 and suppression of NF-κB [40]. So, curcumin could be considered a hopeful agent in targeting AD through Nrf2/Keap1/ARE and interconnected pathways.

#### 4.1.2. Naringenin and Naringin

Naringenin and its glycosylated structure, naringin (naringenin 7-*O*-neohesperidoside), are substantial flavonoids with variant pharmacological effects. The neuroprotective effects of these compounds are repeatedly reported against different neurodegenerative diseases [41]. Naringenin recuperated the streptozotocin-induced AD via enhancing the activity of GPx, SOD, CAT, GST, glutathione reductase (GR), and Na^+^/K^+^-ATPase in a rat model of AD [42]. Also, decreasing the level of MDA in hippocampus has been reported as the main anti-AD approach of naringenin in the animal level [43]. Furthermore, activation of the Nrf2/ARE signaling pathway is a basic protective mechanism of naringenin against the in vivo and in vitro models of neurotoxicity induced by 6-hydroxydopamine (6-OHDA) [44]. Naringenin improved the mitochondrial dysfunction and decreased oxidative stress in brain neurons of Sprague-Dawley rats through interfering with the Nrf2/ARE pathways [45]. In a similar study, naringin could effectively ameliorate the memory deficit in male Wistar rats by improving mitochondrial dysfunction [46]. Naringin also showed a neuroprotective activity against 3-nitropropionic acid (3-NP)-induced neurotoxicity in the PC12 cell line via interfering with the Nrf2 signaling pathway [47]. Furthermore, naringin showed substantial protective effects versus okadaic acid, lipopolysaccharide (LPS), and colchicine-induced dysfunctions via the variant approaches such as mitigating the activity of acetylcholinesterase, increasing the levels of SOD, GSH, CAT and blurting another anti-inflammatory, antioxidant, and antiapoptotic properties [48,49,50]. Overal, naringin and naringenin are promising polyphenols in the management of AD through modulating Nrf2/Keap1/ARE and cross-talk mediators.

#### 4.1.3. Quercetin

Quercetin is a well-known bioflavonoid that can be found in vegetables, fruits and some oils of herbal origin. It was documented that quercetin has a substantial potential to scavenge the ROS, and thereby exert anti-inflammatory, anti-cancer, and neuroprotective activities [28]. In 2017, Fuxing et al. investigated the in vitro advantages of quercetin in neurotoxicity induced by d-galactose (d-gal). Results emphasized that interfering with the Nrf2/ARE signaling pathway is the main neuroprotective mechanism of quercetin that led to improve memory and learning in mice [51]. Moreover, quercetin showed a protective effect in neuronal cultures via enhancing the Nrf2 nuclear translocation, and GSH levels [52]. In another study, a complex of phospholipid and quercetin represented potent antioxidative activities on the retinal pigmented epithelium (ARPE-19 cell line) via activation of the Nrf2 pathway [53]. The manganese-induced neuroinflammation was also properly attenuated via the application of quercetin and interfering with Nrf2/HO-1 and inducible nitric oxide synthase (iNOS)/NF-κB pathways [54]. Quercetin in combination with sitagliptin activated the Nrf2 signaling in a rat model of AD [55]. Besides, the in vivo neuronal toxicity induced by H_2_O_2_ was properly improved by quercetin via decreasing the ROS level [56]. Furthermore, the antioxidant, neuroprotection and anti-inflammatory activities of quercetin on the variant cell lines such as ARPE-19, SH-SY5Y, and APPswe were investigated and approved [57,58].

#### 4.1.4. Chalcones

Chalcones are other important natural compounds, belong to the flavonoid’s family and are present in variant fruits such as apple, citrus, tomato, and several vegetables, for instance, bean sprouts, shallots, potatoes, etc. Diverse biological activities have been reported for chalcones and their derivatives, including neuroprotective, anti-inflammatory, antibacterial, antiviral, antioxidant, anticarcinogenic, antifungal, and antimalarial activities [59]. Xanthohumol is a main chalcone derivative obtained from hop (*Humulus lupulus* L.) that showed anti-inflammatory activities via the induction of HO-1 through the involvement of Nrf2/ARE signaling in microglial BV2 cells [60]. Also, the advantages of novel chalcone derivatives were investigated in the treatment of scopolamine-induced mouse model of learning and memory impairment and results emphasized that chalcone derivatives could properly attenuate the learning and memory impairment through the activation of Nrf2 [61]. Furthermore, 2′,3′-dihydroxy-4′,6′-dimethoxychalcone (DDC) significantly diminished the Aβ induced neuronal death and neurotoxicity on the cortical neuronal cell through enhancing the activation of the Nrf2/ARE pathway and increasing the expression of HO-1 [62].

#### 4.1.5. Other Phenolic Compounds

Some other phenolic compounds have also shown the potential of being used in neurodegeneration and AD. Reducing the inflammatory factors, decreasing ROS generation, enhancing the activity of SOD and neurotrophic factors are some of other anti-AD mechanisms of polyphenolic phytochemicals [63,64,65,66,67]. Rutin is resulted from the glycoside combining the quercetin and the disaccharide rutinose with significant antioxidant and anti-amyloidogenic activities in APPswe cells [58]. As another natural phenol, phloretin improved the amnesia induced by scopolamine in mice via enhancing the activity of antioxidant enzymes, especially CAT, and SOD, and decreasing the level of MDA which all are in near interconnection with Nrf2 [68]. In this line, magnolol is also a lignan that compensated for the learning disability induced by scopolamine via restoring the total nitric oxide synthase and acetylcholinesterase (AChE) activity. Additionally, magnolol improved the antioxidation effects by increasing the SOD activity and decreasing the methane dicarboxylic aldehyde content [69]. Carmona et al. investigated the in vitro anti-AD effects of isoquercitrin, morin, hesperidin, and neohesperidin on MC65, HT22, and APPswe cell lines. These agents prevented the aggregation of Aβ_25–35_, facilitated its disaggregating, decreased the intracellular ROS levels, also attenuated caspase-9, and -3 activations [70]. Pinocembrin and phenethyl ester of caffeic acid are other phenolic compounds that showed a neuroprotective, and anti-AD effects through the Nrf2/HO-1 pathway [71,72,73]. It was documented that luteolin, farrerol, gastrodin, baicalein, and garlic-derived hybrids accomplished their neuroprotective and antioxidant effects by engaging with Nrf2/ARE signaling pathway [74,75,76,77]. Table 1 shows the potential use of phenolic compounds in AD through Nrf2/Keap1/HO-1 and interconnected pathways.

### 4.2. Alkaloids

Alkaloids are nitrogen-containing basic secondary metabolites, divided into true alkaloids (nitrogen-containing heterocyclic compounds) and proto-alkaloids (contain nitrogen atom(s) that is not a part of the heterocyclic ring). Although most of the common alkaloids have been isolated from plants, they could also be found in microorganisms, marine organisms, animals, and fungi. In the plant kingdom, some families contain more alkaloids than others, including Amaryllidaceae, Papaveraceae, Solanaceae, and Ranunculaceae. These organic natural compounds have a wide range of biological and pharmacological activities, including analgesic, anticancer, antimalarial, antioxidant, anxiolytic, anti-inflammatory, antidepressant, antiasthma, antiarrhythmic, antibacterial, and antihyperglycemic effects [28,79,80].

Berberine is an isoquinoline alkaloid with a wide variety of biological and pharmacological effects [81]. It possesses antioxidant and anti-inflammatory properties assessed by in vitro and in vivo models of AD. The regulatory effects of berberine on Nrf2/ARE pathway could be considered as one of its main protective mechanisms in oxidative stress-induced neuronal cell damages. In some in vitro studies, pretreatment with berberine significantly improved SOD activity and intracellular GSH levels, while decreased ROS generation, MDA formation, and LDH release [81,82,83]. Sadraie et al. reported that berberine improved spatial recognition memory in LPS-induced learning and memory dysfunctions in rats by up-regulation of Nrf2 target genes, like SOD and CAT. It also improved antioxidant capacity through increasing GSH and GPx levels and decreasing MDA and protein carbonyl levels [84]. Oxidative stress, in turn, could activate inflammatory pathways with a major contribution to the pathogenesis of AD. Furthermore, Nrf2 can decrease the transcription of pro-inflammatory cytokines in microglia, astrocytes, and macrophages; and increase the expression of anti-inflammatory mediators [85]. Besides, berberine also attenuated inflammation-related indices like TNF-α, IL-6, NF-κB, and toll-like receptor 4 (TLR4) [84,86]. Moreover, de Oliveira and colleagues indicated similar protective effects from berberine on streptozotocin-induced dementia in male Wistar rats [87].

Trigonelline (TRG) is another alkaloid that showed anti-AD activities in Aβ_1–40_- and LPS-induced AD, in vivo [88,89]. TRG improved spatial learning and memory in the Morris water maze and Y maze test. According to a cross-talk between antioxidant and anti-inflammatory pathways, neuroprotective effects of TRG in AD could be related to its regulating role in ARE and Nrf2 pathway (Table 2).

Tetramethylpyrazine, also known as ligustrazine, is an alkaloid isolated from Chinese herbal medicine *Ligusticum wallichii* Franchat, showed considerable in vitro and in vivo anti-AD effects on cobalt chloride-induced neurotoxicity in PC12 cells and male Wistar rats. This effect was exerted through the stimulation of Nrf2/glutamate-cysteine ligase (GCL)-mediated regulation of GSH, GSSG, and repression of hypoxia-inducible factor-1α (HIF-1α)/NADPH oxidase (NOX)-mediated ROS production and superoxide level, contributed to the amelioration of oxidative stress and then increasing cell viability under hypoxic conditions [90]. Ligustrazine phosphate (LP) is the synthetic product of ligustrazine that exerts considerable effects on scopolamine-induced amnesia in male rats. LP enhanced the activities of the antioxidant enzymes (SOD and GPx activities) with a remarkable reduction in lipid peroxidation levels [91,92]. The LP transdermal ethosomal system had a higher penetration ability than aqueous one [91]. Shi et al. also showed its higher ability in improving behavioral performance when used in combination with huperzine A, a sesquiterpene alkaloid that extracted from Chinese club moss *Huperzia serrata* [92]. Besides, another study represented that a cholinesterase inhibitor alkaloid, huperzine A, prevented morphological damages and increased cell viability via regulating AREs [93].

Piperine, as the most abundant alkaloid in pepper, has several pharmacological activities like anticonvulsant, anti-depressant and improving cognitive abilities [94,95]. Yang et al. synthesized a novel piperine derivative, HJ22, in attenuating cognitive impairment, oxidative stress, neuroinflammation and apoptosis in rats. HJ22 was connected to the Keap1, and prevented from protein-protein interaction of Keap1/Nrf2 complex, thereby nuclear Nrf2, ARE and downstream genes like SOD, CAT, and GR expression in the hippocampus of rats. In addition, activation of Nrf2, significantly decreased the expression of thioredoxin-interacting protein (TXNIP), contributing to the inactivation of nod-like receptor protein 3 (NLRP3) inflammasome, and IL-1β depletion [94].

Dauricine, an isoquinoline alkaloid that isolated from the *Rhizoma menispermi*, has been found to yield neuroprotective effects. Wang et al. reported that dauricine increased cell viability, decreased Aβ_1–42_ secretion, alleviated the chronic and acute oxidative damages, and repressed the apoptotic rate in AD models. These effects were in a near link with the regulation of the Nrf2/Keap1/Bcl-2 pathway [96]. As a bisbenzylisoquinoline alkaloid, fangchinoline, has been shown to protect HT-22 mouse neuronal cells against glutamate-induced oxidative damage through the up-regulation of Nrf2 and its target genes, HO-1 and SOD. Fangchinoline also down-regulated Keap1 expression. These mechanisms led to an increase in cell viability and blocked cell morphological damages [97]. Deoxyvasicine is a main quinazoline alkaloid isolated from the aerial parts of *Peganum harmala* Linn. It effectively ameliorated learning and memory deficits in scopolamine-treated mice by the same mechanisms as huperzine A did in attenuating oxidative stress and neuroinflammation [98].

Plumbagin is another alkaloid that isolated from the plants of Plumbago genus. It has been reported that plumbagin indicated its anti-Alzheimer effects through up-regulating of Nrf2/ARE pathway. Plumbagin also prevented the cognitive impairments induced by STZ in mice via Nrf2/ARE mediated attenuation of astrogliosis and suppression of the β-secretase enzyme [99].

As another alkaloid, embelin (2,5-dihydroxy-3-undecyl-1,4-benzoquinone) notably improved the memory retention and recognition index in scopolamine-induced amnesia in rats by elevated expression of SOD1 and CAT as Nrf2 target genes [100]. Embelin also increased the expression of brain-derived neurotrophic factor (BDNF), as an inducer of Nrf2 [100,101]. Harmaline and harmine isolated from *Peganum harmala* L. also showed promising anti-amnesic effects on the scopolamine-induced memory deficits in mice [102]. Pretreatment with these β-carboline alkaloids remarkably improved SOD and GPx activities, while relieved MDA and TNF-α levels [102].

In the same studies on alkaloids, isorhynchophylline, oxindole [103], aloperine [104], matrine, methyl jasmonate, neferine, norcepharadione B, and vincamine [105,106,107,108,109] showed remarkable anti-Alzheimer properties mediated by improving antioxidant capacity and targeting Nrf2/ARE pathway.

Recent studies also showed that *Corydalis edulis* Maxim. has several pharmacological activities. Hence, Liang and colleagues evaluated the efficacy of *Corydalis edulis* Maxim. total alkaloids (CETA) in d-gal-induced AD in rats. In their study, eleven alkaloids (protopine, berberine hydrochloride, berberine, dehydrocorydaline, acetylcorynoline, fumariline, tetrahydroberberine, tanguinarine, ochotenimine, palmatine chloride, and corynoline) were identified by UPLC-MS/MS analysis from CETA extract [110]. In another study, the effects of a total alkaloidal extract from *Murraya koenigii* (MKA) leaves (girinimbine, mahanimbine and murrayanine) was evaluated on age-related oxidative stress in aged mice [111]. CETA and MKA both ameliorated oxidative stress via the Nrf2-dependent induction of SOD and CAT and suppressing NF-κB expression, TNF-α, IL-1β levels and Aβ accumulation [85,110,111].

Therefore, secondary metabolites mentioned in this part could be promising for drug development in preventing AD and related diseases. Other mechanisms of these compounds are mentioned in Table 2.

### 4.3. Terpenes and Terpenoids

Terpenes are known as an important group of secondary metabolites composed of the numbers of isoprene (C_5_H_8_) units. Isoprene is an unsaturated hydrocarbon that could be constructed by disparate plants and animals. Modifying various functional groups, as well as rotating, removing, or oxidizing the methyl group at variant positions of primitive structure of terpenes, consequences to the terpenoid category. Antibacterial, antioxidant, antifungal, anti-inflammatory, anticarcinogenic, neuroprotective, and cardioprotective activities are some of the important pharmacological effects of terpenes and terpenoid compounds [112].

It was reported that in vitro and in vivo neuronal impairment induced by ethanol, was properly improved via the antiapoptotic and antioxidative activities of carvacrol [113]. 1,8-cineole and α-pinene are other monoterpenes agents, which demonstrated an in vitro neuroprotective activity against the oxidative stress induced by H_2_O_2_ via the interposition to the ROS production and increasing the level of several enzymes and compounds like CAT, SOD, GPx, and HO-1 [114]. The investigation from the hippocampus of Swiss mice treated with *p*-cymene emphasized that this monoterpene reduced nitrite content, lipid peroxidation, and enhanced the activity of CAT and SOD [115]. Similarly, linalool is a monoterpenoid that showed a protective effect against the in vivo cognitive deficits via enhancing the activity of GPx, SOD, and the Nrf2/HO-1 pathway proteins [116]. The in vivo cognitive deficits mediated by a high-fat diet, after the administration of thymol, was satisfactorily improved via the up-regulating the Nrf2/HO-1 pathway [117]. Similarly, the neuroprotective and anti-Alzheimer’s effects of other monoterpenes have been repeatedly mentioned in various studies. Enhancing the activity of antioxidant enzymes and up-regulation of Nrf2 are the main neuroprotective mechanisms of carvacryl acetate, borneol, and geraniol [118,119,120].

7β-(3-ethylcis-crotonoyloxy)-1α-(2-methylbutyryloxy)3,14-dehydro-*Z*-notonipetranone (ECN) is a neuroprotective sesquiterpenoid that has previously shown anti-inflammatory and cytoprotective effects through activating the Nrf2/HO-1 signaling pathway and decreasing ROS generation in vivo and in vitro studies [121,122]. Administration of some other sesquiterpenoids, including lactucopicrin [123], α-cyperone [124], and artemether [125] ameliorated the oxidative stress via activation of the Nrf2 pathway and downstream mediators such as HO-1, SOD, and anti-inflammatory mediators in the mice model of AD. Park and colleagues showed, bakkenolide B (a sesquiterpene), isolated from *Petasites japonicus* leaves, might be considered a strategy in the treatment/prevention of neurodegenerative diseases like AD. Bakkenolide B, exerted its neuroprotective effects by up-regulation of Nrf2/ARE signaling pathways and related downstream factors, including HO-1 and NQO1, which led to reduced ROS production and neuroinflammation. These activations were associated with the enhancement of AMPK phosphorylation [126].

Carnosic acid is an important diterpene with various biological activities such as anti-inflammatory, neuroprotective, antioxidant, and anticancer effects. Carnosic acid protected the SH-SY5Y cell line against neurotoxicity via interfering with the PI3K/Akt/Nrf2 signaling pathway [127]. Moreover, it was documented that Nrf2 plays a critical role in the neural differentiation and neuroprotective effects mediated by carnosic acid [127,128]. Takumi et al. investigated the in vivo and in vitro neuroprotective effects of carnosic acid, and protecting the PC12h cell line via activating the Nrf2/ARE pathways [129]. Furthermore, carnosic acid diminished the production of Aβ_1–42_ in SH-SY5Y cell lines through inducing the activation of TACE, expressions of Aβ-degrading enzymes, and a poor modulatory effect on Nrf2 [130]. As another lactone diterpenoid, andrographolide, that isolated from *Andrographis paniculata* showed in vitro anti-Alzheimer’s advantages in HT22 and PC12 cell lines via activation of Nrf2/ARE/HO-1 and the Nrf2-mediated p62 signaling pathways [131,132].

As another terpene, compound K is a triterpenoid structure isolated from red ginseng that could significantly improve memory functions in an animal model of neurotoxicity. Furthermore, interfering with the Nrf2 signaling pathway and antioxidant enzymes was suggested as a main neuroprotective mechanism of compound K [133]. It was documented that ginsenoside showed its neuroprotective effects via activation of Nrf2 and inhibiting the ROS/ASK-1 in SH-SY5Y cell lines [134]. Tom et al. designed a study to investigate the anti-Alzheimer effects of gedunin in the IMG cell line. The results emphasized that gedunin prevented neurotoxicity via interfering with the Nrf2 and NF-κB signaling pathways [135]. Besides, administration of lycopene leads to attenuate the cognitive impairments and amyloidogenesis induced by LPS through inhibiting oxidative stress and neuroinflammation [136]. In another study by Xiangbao et al. gypenoside XVII effectively attenuated the neurotoxicity induced by Aβ_25–35_ via activation of Nrf2/ARE pathways [137]. Table 3 indicates the potential of terpenes/terpenoids against AD through Nrf2/Keap1/ARE.

### 4.4. Carotenoids

Carotenoids are lipophilic and richly colored molecules that are found in a wide variety of plants, algae, and bacteria. They are responsible for the red, yellow, and orange colors of many plants. Carotenoids are divided into two major classes of xanthophylls (contain at least one oxygen) and carotenes (hydrocarbons without oxygen). Several studies showed antioxidant, anti-inflammatory and antiapoptotic activities of carotenoids, thus they play effective roles in neurodegeneration. Although over 1100 various carotenoids have been yet identified, researchers have focused mainly on a few of them with their beneficial effects on human health [138,139,140]. Astaxanthin (AST) is a xanthophyll keto-carotenoid with significant antioxidant properties, and because of its structure, it could be able to pass through the blood-brain barrier (BBB) [141,142,143]. AST conserved HT-22 mouse neuronal cells from glutamate-induced ex situ neurotoxicity, via Nrf2/ARE-dependent HO-1 expression [144]. In an in vivo study, Al-Amin et al. reported that AST ameliorated scopolamine-induced memory impairment in mice. This result could be attributed to the up-regulation of Nrf2 target genes, like SOD and CAT [145]. Moreover, with relatively similar mechanisms, Taksima and colleagues showed that AST orally administration improved spatial learning and memory in Aβ_1–42_-induced AD in rats [146]. Later, in vitro and in vivo studies demonstrated that AST significantly reduced ROS, thiobarbituric acid levels while elevated GSH and GSH/GSSG ratio as protective antioxidants against oxidative stress. Besides, because of the cross-talk between Nrf2 and anti-inflammatory pathways, AST indicated a remarkable reduction in the expression of the proinflammatory cytokines like TNF-α, IL-1β and IL-6 [147,148], as well as upstream mediators (e.g., Akt, *p*-ERK) [149]. In an in vitro study, Yang et al. indicated that AST attenuated tert-butyl hydroperoxide-induced production of ROS in PC12 cells. They also reported that the mentioned neuroprotective effects were enhanced in a combination use of AST with huperzine A [93]. These results confirmed AST potential efficacy in managing and/or treating AD. Following the anti-Alzheimer effects of carotenoids, the in vitro and in vivo investigation of a xanthophyll carotenoid, crocin, indicated its neuroprotective effects via regulation of oxidative stress-associated apoptosis signaling pathway [150]. Studies reported that crocin protected HT-22 mouse neuronal cells against L-glu-induced damages by an increase in phosphorylation levels of Akt, which led to the enhancement of Nrf2/ARE-dependent protection in the oxidative stress pathway [150,151]. They also indicated that crocin could improve spatial learning and memory in an AlCl_3_/d-gal-induced AD in mice by increasing GPx and SOD activities [150]. In another study by Mohammadzadeh *et al*., crocin antagonized malathion-induced cognitive deficit in rats. This neuroprotective effect of crocin was attributed to its antioxidant activities through increasing GSH, decreasing MDA levels, thereby, TNF-α, IL-6, and repressing tau hyperphosphorylation [152]. Also, it was reported that crocin ameliorated streptozotocin-induced spatial memory deficit via similar antioxidative mechanisms [153,154].

Lycopene, a tetraterpene carotene, is a red plant pigment found in tomatoes, watermelons, grapefruit, etc, which has exerted significant antioxidant activity in recent studies [136,155]. Wang et al. found that pretreatment with lycopene prevented LPS-induced AD in the preclinical studies by increasing the expression of nuclear Nrf2, HO-1, NQO-1, SOD, CAT, and GSH in Nrf2/ARE antioxidant pathway. Additionally, this activation of Nrf2-dependent target genes could relieve NF-κB nuclear translocation and elevated expression of anti-inflammatory mediators like IL-10 [85,136]. Besides, lycopene enhanced spatial and passive memory of tau transgenic mice through increasing in antioxidant capacity [156]. β-carotene and levocarnitine are also some of the other carotenoids with anti-Alzheimer effects on mice related to the involvement of the Nrf2/ARE pathway [157,158].

Strigolactone is a novel emerged apocarotenoid plant hormone [159]. Kurt and colleagues indicated that GR24rac, a strigolactone analogue, could exert glia/neuroprotective effects on LPS-treated mouse microglial cells. GR24rac showed that these effects are concomitant with an increase in nuclear Nrf2, HO-1, and NQO-1 expression, which could play a role in reducing TNF-α and IL-1β levels. The activation of Nrf2/ARE pathway resulted in inhibition of NF-κB nuclear deposition induced by LPS. In addition, NF-κB affected on COX-2 and iNOS levels. Consequently, these sequences caused neuroprotective and anti-neuroinflammatory properties of GR24rac [160].

The overall studies suggest carotenoids as a promising source for the management or treatment of Alzheimer’s and related diseases. Other mechanisms of these secondary metabolites involved in their effects are given in Table 4.

### 4.5. Sulfur-Containing Secondary Metabolites

Sulfur-containing secondary metabolites are known as an essential class of plant secondary metabolites, with a limited number of identified compounds (approximately 200). Onion and garlic volatile components, as well as glucosinolate’s agents, are considered as two major groups of this category that observed in high levels in plants. Glucosinolate’s components and their breakdown products such as oxazolidinethiones, epithionitriles, isothiocyanates, and thiocyanates are the most known group of this secondary metabolite [161,162]. 1,3-dithiolthiones, mono and disulfide derivatives, and cysteine sulfoxide are natural constituents of onion and garlic volatile components [162]. A wide range of different biological effects, such as antiasthmatic, antibacterial, antioxidant, anticarcinogenic, antithrombotic, antihyperlipidemic, and antiangiogenic activities, have been observed and reported from these compounds. In addition, several structures of sulfur-containing metabolites showed suitable advantages to the management or prevention of various neurodegenerative diseases, especially AD [28,162].

#### 4.5.1. Sulforaphane

Sulforaphane, with the molecular formula of C_6_H_11_NOS_2_, is an isothiocyanate agent that belongs to the organosulfur compounds. Due to its isothiocyanate group, sulforaphane has achieved electrophilic properties to allow this compound in interacting with a nucleophiles structure such as specific protein’s residues like cysteine [163]. Sulforaphane showed significant anti-inflammatory and antioxidative activity in an in vitro model of AD via up-regulating the expression of Nrf2 [164]. Additionally, the administration of sulforaphane leads to the enhancement of total GSH level and GST in the SH-SY5Y cell line [165]. Sulforaphane properly protected hippocampal neurons versus hemin induced neurotoxicity via reinforcing antioxidant defense approaches and activating the ARE/Nrf2 pathway [166]. In a similar study, neuronal cell isolated from the Wistar rat’s striatum, protected from paraquat and H_2_O_2_-induced toxicity via the administration of sulforaphane and an analog of isothiocyanate [167]. Moreover, the neuroprotective advantages of sulforaphane in the in vivo and in vitro models of PD have been proved in several studies [168,169,170]. Furthermore, it exhibited a suitable anti-Alzheimer activity against Aβ peptide via interfering with Nrf2/HO-1 cascade, which leads to the attainment of anti-inflammatory properties in human THP-1 macrophages [171]. In a recent study, the efficiency of sulforaphane, in the protection of mice with Alzheimer’s-like lesions was investigated by up-regulating Nrf2 transcription activity [172]. Furthermore, sulforaphane protected several cells such as astrocytes and PC12 cells against neurotoxicity and oxidative stress via the activation of Nrf2 and other related enzymes [173,174].

#### 4.5.2. *S*-Allyl Cysteine

*S*-Allyl cysteine is a sulfur-containing secondary metabolite with the chemical formula of C_6_H_11_NO_2_S that can be found in significant amounts in fresh garlic. *S*-allyl cysteine is a cysteine derivative obtained by adding an allyl group to the sulfur atom. Various biological effects of this compound such as the antihyperlipidemic, antioxidant, neuroprotective, antihepatotoxic, anticancer, and chemopreventiveactivities, were documented [175]. *S*-allyl cysteine suppressed oxidative stress, GPx, and GSH in a mouse model of AD [175]. Furthermore, *S*-allyl cysteine showed in vitro and in vivo antioxidant, cytoprotective, neuroprotective, and anti-amyloidogenic effects through attenuating several signaling pathways and enzyme levels like MDA, SOD, Nrf2, etc. [176,177,178]. Similarly, the investigation of the beneficial effect of *S*-allyl-l-cysteine and isoliquiritigenin in PC12 cell lines demonstrated that these compounds improved the mitochondrial membrane potential [179].

Hippocampal and cerebellar granule neurons isolated from embryos of Wistar rats were protected by *S*-allyl-l-cysteine against the neuronal toxicity induced by Aβ protein [180]. *S*-allyl, *S*-ethyl, and *S*-propyl are three other cysteine amino acid-containing metabolites, which have been shown to reduce the production of Aβ protein, diminished the activity of SOD, GPx and CAT in the brain of mice treated with d-gal [181].

#### 4.5.3. Other Sulfur-Containing Secondary Metabolites

6-(Methylsulfinyl)hexyl isothiocyanate is another sulfur-containing structure that satisfactorily protected the studied animals against the Aβ-induced oxidative stress, cognitive deficit, and inflammation [182]. The in vitro cytotoxicity induced by H_2_O_2_ was attenuated via allicin through the regulating of ROS levels [183]. Allicin improved the aging cognitive deficits in male C57BL/6 mice via the activation of Nrf2 signaling pathways [184]. Also, interfering with *p*-ERK/Nrf2 signaling pathway is the main mechanism of allicin to protect models of AD against the endoplasmic reticulum stress-related cognitive deficits [185]. Thiacremonone, 3*H*-1,2-dithiole-3-thione, hydrogen sulfide and lipoic acid are some of the other sulfur-containing secondary metabolites with proven neuroprotective and antioxidant effects on various in vitro and in vivo models of AD [186,187,188,189]. Table 5 indicates the potential of Sulfur-Containing Secondary Metabolites against AD through Nrf2/Keap1/ARE.

### 4.6. Miscellaneous Compounds

Several miscellaneous secondary metabolites have also demonstrated promising anti-Alzheimer effects. Ginsenosides, also called ginseng saponins, are one of the major active components of *Panax ginseng* classified into protopanaxadiol saponin and protopanaxatriol saponin. Protopanaxadiol saponin metabolized through gut microflora into the ginsenoside compound K (20-*O*-β-d-glucopyranosyl-20(*S*)-protopanaxadiol) (CK) [133,190]. CK showed significant neuroprotective effects in preclinical studies via increasing the expression of Nrf2/Keap1/ARE signaling pathway-related factors such as nuclear Nrf2, HO-1, NQO1, SOD, and GPx. In addition, CK attenuated the expression of Keap1 and MDA levels. Collectively, these effects led to a decrease in Aβ expression, the number of apoptotic cells and an improvement in spatial learning, cognitive and memory function [190,191]. Hence, these studies suggested that CK could be a promising agent in the prevention and treatment of AD.

Ginsenoside Rd (GRd) is a protopanaxadiol type ginsenosides [192]. As well as CK, GRd exerted its neuroprotective effects against Aβ_25–35_-induced neuronal damage in primary cultured hippocampal neurons via the regulation of Nrf2 target genes in the oxidative stress pathway [193]. 20(*S*)-protopanaxatriol (PPT) is another ginsenoside that showed beneficial effects in the central nervous system. Thus, Lu et al. evaluated its neuroprotective effect in scopolamine-induced cognitive deficits in male mice. Like protopanaxadiol ginsenosides, PPT could improve memory and learning abilities of mice in several behavioral tests, by suppressing oxidative stress and increasing cholinergic neurotransmission [194]. Consistently, ginsenoside Rg1 (Rg1) is a protopanaxatriol type ginsenosides that abundantly contained in ginseng [192]. Rg1 improved chronic stress-induced learning and memory impairments in mice through reducing the ROS production, MDA and 8-OHdG levels while increased the SOD activity. In addition, this regulation of Nrf2/ARE-dependent factors could inhibit NOX2 expression that is also involved in the Rg1 mechanisms of action [195,196].

Pseudoginsenoside-F11 (PF11), an ocotillol-type saponin, mitigated learning and memory deficits in Aβ_1–42_-induced AD in mice. PF11 also restored SOD and GPx activities and reduced Aβ precursor protein (APP) expression MDA production in the cortex of APP/PS1 mice [197]. As another saponin, timosaponin B-II attenuated scopolamine-induced cognition deficits through increasing SOD, GPx, and decreasing MDA [198].

Consistently, anthraquinones, lactones, vitamins, fatty acids, and naphthoquinone pigments are other miscellaneous compounds. In this line, an in vitro and in vivo investigation of aloe-emodin, an anthraquinone compound, showed its neuroprotective effects by modulating oxidative stress. Aloe-emodin significantly reduced intracellular ROS accumulation, NO, MDA levels while elevated SOD and GPx activity as protective factors against both models of H_2_O_2_-induced cytotoxicity in PC12 cells and scopolamine-induced amnesia in mice [199]. Besides, Fragoulis and colleagues reported that methysticin, a kavalactone, indicated its neuroprotective effects on 52-weeks old transgenic mice via Nrf2-dependent HO-1 expression. Besides, activation of Nrf2/ARE pathway relieved TNF-α, IL-17A, microgliosis/astrogliosis, and improved long-term memory impairment of APP/Psen1 mice [200].

On introducing vitamins with promising antioxidative effects in AD, α-tocopherol (vitamin E) decreased oxidative stress by up-regulating the expression of Nrf2 and reducing in iNOS levels. Besides, α-tocopherol induced the expression of genes participated in the processing of APP and modulating the expression of genes participated in autophagy. Thus, it could be able to decrease the neurotoxicity induced by Aβ_1–42_ in retinoic acid-differentiated neuroblastoma SH-SY5Y cells [201]. As Wang et al. indicated, α-tocopherol quinine (α-TQ), an oxidative metabolite of α-tocopherol, ameliorated biochemical and behavioral changes in vitro and in vivo. They found that α-TQ decreased ROS production and MDA levels and increased SOD activity as Nrf2 target genes in the brain of transgenic AD mice. Thereby, α-TQ reduced NF-κB activation, iNOS, IL-1β and IL-6 expression, also inhibited microglia activation regarding improving spatial cognitive performance in AD mice [202].

Of fatty acids, α-linolenic acid (ALA), an omega-3 polyunsaturated fatty acid that is present in vegetable oils, possesses potent neuroprotective and anti-inflammatory properties [203,204]. ALA represented its antioxidant activities through inducing Nrf2 and HO-1 expression, thereby has been suggested as a promising source for combating AD [203].

As another miscellaneous compound, shikonin, a naphthoquinone pigment, extracted from the roots of *Lithospermum erythrorhizon*, and indicated several biological and pharmacological properties, like antioxidant, anti-inflammatory, antimicrobial, antiviral, antithrombotic, and cancer-preventing effects [205,206]. Tong and colleagues evaluated the neuroprotective effects of shikonin against neuronal insults induced by Aβ_1–42_ in PC12 cells. Shikonin significantly ameliorated Aβ_1–42_-induced oxidative stress by reducing the ROS production, MDA level and LDH release, and increasing the levels of SOD, CAT and GPx. Moreover, improving the antioxidant capacity could indirectly enhance cell viability by regulating apoptotic factors [207]. Among other miscellaneous pigments, betalains are a class of red/yellow tyrosine-derived pigments, where they could be replaced by anthocyanin pigments in plants. Betalain-enriched extracts have been found to possess potential inhibitory effects on acetylcholine esterase, and oxidative stress. So, these compounds could be also of great importance in combating AD [208]. These compounds are potential antioxidants capable of reverting oxidative stress through modifying the expression of Nrf2 [209]. In this line, melatonin with the potential of pigmentation, greatly activated Nrf2, thereby counteracted LPS-Induced oxidative stress and rescued postnatal rat brain [210].

In an in vitro study, Khodagholi et al. indicated that pretreatment with chitosan, an oligosaccharide, significantly improved Nrf2 activity, HO-1 expression, GSH concentration, γ-glutamylcysteine (γ-GCS) levels, and Hsp-70 while decreased NF-κB, caspase-3, and Aβ formation. The regulating properties of chitosan on Nrf2/ARE pathway and its related target genes could be considered as one of the main protective mechanisms of chitosan in the management of AD [211]. Additionally, *Lycium barbarum* polysaccharide [212], *Amanita caesarea* polysaccharides [213], and *Inonotus obliquus* polysaccharides [214] showed significant neuroprotective effects mediated by targeting Keap1/Nrf2/ARE signaling pathway and their related factors (Table 6). Therefore, these secondary metabolites with known mechanisms of action could be a promising source for drug development in preventing or treating AD and related diseases. Other pharmacological mechanisms of these secondary metabolites involved in their effects are given in Table 6.

## 5. Clinical Complementary Uses of Plant Secondary Metabolites in Cognitive Dysfunctions

Plant-derived secondary metabolites have shown beneficial effects on human health along with promising roles in the prevention, management, and treatment of AD. Based on their antioxidant effects, several clinical trials are trying the possible therapeutic effects of phytochemicals in AD. Some plants rich in polyphenols of anthocyanin class meaningfully improved some aspects of cognition in healthy old adults, through reducing oxidative stress (e.g., nitrite, and iNOS) and inflammatory markers (e.g., COX-2, and TNF-α) [216]. In this line, anthocyanin-rich blueberry improved working memory through increasing perfusion/activation of brain areas related to cognitive function in healthy older adults [217]. In a randomized cross-over study, a mixed beverage of anthocyanins improved cognitive functions in healthy older adults [218]. These secondary metabolites also improved cognitive function and brain metabolism and significantly affected the early stages of AD in a double-blinded placebo-controlled pilot study [219]. In a double-blind, placebo-controlled, crossover investigation by Kennedy et al., a single dose orally administration of resveratrol modulated cerebral blood flow and cognitive performance [220].

In addition to the phenolic compounds, some other secondary metabolites have also shown a bright future towards the improvement of cognitive dysfunction. Supplementation with carotenoids significantly attenuated memory dysfunction during a 12-month randomized, double-blind placebo-controlled clinical trial [221]. Besides, an increased intake of carotenoids in patients with mild cognitive impairment was helpful in lowering the risk of conversion to dementia, GPx and SOD [222]. Some other clinical trials are also ongoing to evaluate the possible potentials of phytochemicals in AD.

## 6. Conclusions and Perspectives

Compelling evidence has shown the key destructive role of oxidative stress in the pathogenesis of AD, along with the critical role of Nrf2/ARE in ameliorating neuronal/cognitional complications. The secondary metabolites of natural sources have found to be promising agents in targeting the aforementioned pathways/mediators in AD, possessing more efficacy/potency while lower side effects. In this regard, phenolic compounds, alkaloids, terpene/terpenoids, carotenoids, sulfur-compounds, as well as some other plant-derived miscellaneous compounds have been accordingly introduced as multi-target compounds in modulating several dysregulated mediators, especially those with a near interconnection with Nrf2/Keap1/ARE and related apoptotic/inflammatory pathways. In cognitive dysfunction, the aforementioned antioxidative pathway, seems to be in the upstream of either apoptotic (Bax and caspase) and inflammatory (TNF-α and ILs) mediators. So, attenuating Nrf2/Keap1/ARE could play a pivotal role in combating AD. Several clinical trials have also been provided to evaluate the therapeutic potential of phytochemicals based on their antioxidant activity. Despite their effectiveness, plant secondary metabolites often suffer from some pharmacokinetic limitations, including poor bioavailability, low solubility/selectivity, and week absorption rate, which urges the needs for developing novel delivery systems [223].

Such studies will provide novel applications of plant-derived secondary metabolites in the prevention, management, and treatment of AD, by stimulating antioxidant mediators and suppressing oxidative pathways. Additional studies are also required to reveal the precise role of Nrf2/ARE and interconnected mediators in AD, and the ways to be targeted by potential phytochemicals in well-controlled clinical trials.

## Figures and Tables

**Table 1:** Polyphenols in combating AD through Nrf2/Keap1/ARE and interconnected pathways.

Compounds	Types of Study	Cell Line(s)/Animal Model(s)	Mechanisms of Action	References
Curcumin	In vitro	human neuroblastoma cell lines (SH-SY5Y, IMR-32)	↑APE1 and Nrf2	[32]
	In vitro, In vivo	human neuroblastoma cell lines (SH-SY5Y), CD1 athymic mice	↓caspase-3 and caspase 7 ↓H_2_O_2_ ↑GSH ↑the ratio of free to oxidized GSH	[34]
	In vivo	male Sprague-Dawley rats	↓mitochondrial dysfunction ↓MDA ↓ROS ↑Thiol	[35]
		male AD model (APP/PS1dE9) mice	↓IL-6 ↓ROS ↓Aβ level ↓TNF-α ↑SOD activities	[36]
		male ICR mice model of traumatic brain injury	↓neuronal apoptosis ↓MDA ↑SOD ↑GPx	[38]
		Japanese quails (*Coturnix coturnix* japonica)	↓MDA levels ↑SOD ↑GPx ↑CAT ↑Nrf2 and HO-1 levels	[39]
Curcumin and berberine	In vivo	B6C3-Tg (APPswePSEN1dE9)/Nju double transgenic mice model of AD	↓IL-1β ↓TNF-α ↓IL-6 ↓GFAP and IBA1	[37]
	In vitro	human neuroblastoma cell line (SH-SY5Y)	↓ROS ↓iNOS, ↓NO ↓NF-κB ↑Nrf2	[40]
	In vitro	rat adrenal pheochromocytoma cell line (PC12)	↑Nrf2/HO-1 protein expression ↓Keap1 ↑translation of Nrf2 into nuclear ↑SOD/CAT ↑Bcl-2 ↓Bax and cytochrome c	[33]
Naringenin	In vivo	male Wistar rats’ model of AD	↑SOD ↑GPx ↑GST ↑CAT ↑GR ↑Na^+^/K^+^ ATPase	[42]
			↓MDA levels	[43]
	In vivo, In vitro	male C57BL/6 mice model of PD, human neuroblastoma cell line (SH-SY5Y)	↑GSH levels ↑Nrf2/ARE pathway ↑ARE pathway genes ↓ROS formation ↑Nrf2 protein levels	[44]
	In vitro	neurons isolated from the brains of Sprague-Dawley rats	↓ROS ↑high-energy phosphates level ↑mitochondrial ANT transport activity ↑mitochondrial membrane potential ↑expression of Nrf2 ↑activation of the Nrf2/ARE	[45]
Naringin	In vivo	male Wistar rats’ model of AD	↑CAT ↑SOD ↑GSH ↓TNF-α ↓IL-1β	[46]
	In vitro	pheochromocytoma cell line (PC12)	↓LDH ↓lipid peroxidation ↓ROS generation ↑GPx ↑CAT ↑GR ↑SOD ↑GSH levels ↑Nrf2 activation ↑HO-1 and NQO-1	[47]
	In vivo	male Wistar rats’ model of cognitive impairment	↑CAT ↑SOD ↑GSH levels ↓TNF-α, ↓TGF-β, ↓IL-1β ↓NF-κβ p65 subunit, ↓caspase-3	[48]
	In vitro	pheochromocytoma cell line (PC12)	↓CYP2E1 ↓ROS rectify the antioxidant protein contents of Nrf2, HO-1, SOD2, and GSS	[49]
	In vivo	male Wistar rats’ model of cognitive impairment	↓MDA ↓nitrite ↑CAT ↑SOD ↑GST ↑GSH levels	[50]
Quercetin	In vivo	male Kunming strain mice model of brain aging process and learning and memory defect	↑Nrf2/ARE ↑Nrf2 ↑HO-1 ↑SOD	[51]
	In vitro	cerebellar granule neurons isolated from Sprague-Dawley rats	↑GCLC gene expression ↑activation of the Nrf2 ↑GSH	[52]
	In vitro	human RPE cell line (ARPE-19)	↑GPx ↓ROS and MDA ↑HO-1, NQO-1, and GCL ↑CAT ↑SOD ↑Nrf2	[53]
	In vitro, In vivo	Sprague-Dawley male rats, Neuroepithelioma cell line (SK-N-MC)	↑CAT ↑SOD ↓MDA ↑GSH levels ↓ROS	[54]
	In vivo	male Sprague-Dawley rats	↑CAT ↑SOD ↓MDA ↑GSH levels ↑Nrf2/HO-1 pathway	[55]
	In vitro, In vivo	homozygotic transgenic mouse line B6.129S7-Sod2tm1Leb/J, hippocampal neurons isolated from Sprague-Dawley rat embryos	↓ROS	[56]
	In vitro	human RPE cell line (ARPE-19)	↑Nrf2 ↑HO-1	[57]
		APP695-transfected SH-SY5Y cell line (APPswe), human neuroblastoma cell lines (SH-SY5Y)	↓lipid peroxidation ↓MDA ↑GSH levels ↓ROS	[58]
Xanthohumol	In vitro	mouse microglia BV2 cells	↑Nrf2-ARE signaling pathway activation ↑Nrf2 expression ↑HO-1 expression ↑GSH ↓NO, IL-1β, and TNF-α ↓NF-κB	[60]
Chalcone derivative	In vivo	scopolamine-induced mice model	↑Nrf2/HO-1 protein expression ↓ROS ↑Superoxide dismutase activity	[61]
2′,3′-Dihydroxy-4′,6′-dimethoxy-chalcone (DDC)	In vitro	cerebellar cortex neurons isolated from embryonic 17–19-day-old Wistar/ST rat fetuses	↑Nrf2-ARE signaling pathway activation ↑Nrf2 expression ↑HO-1 expression	[62]
Rutin	In vitro	APP695-transfected SH-SY5Y cell line (APPswe), human neuroblastoma cell lines (SH-SY5Y)	↓lipid peroxidation ↓MDA ↑GSH levels ↓ROS	[58]
Phloretin	In vivo	scopolamine-induced mice model	↑CAT ↑SOD ↓MDA	[68]
Magnolol			↑total nitric oxide synthase ↑AChE activity ↑SOD ↓methane dicarboxylic aldehyde	[69]
Morin	In vitro	mouse hippocampal nerve cells (HT22), Swedish mutant APP stable cell line (APP695-transfected SH-SY5Y)	↓ROS levels ↓caspase-9, and -3 ↓β- and γ-secretase	[70]
Isoquercitrin			↓ROS levels ↓caspase-9, and -3 ↓β- and γ-secretase	[70]
Pinocembrin	In vitro	human neuroblastoma cell lines (SH-SY5Y)	↑Nrf2 protein levels ↑Nrf2/HO-1 pathway ↓ROS levels	[71]
Caffeic acid derivative	In vivo, In vitro	male Kunming mice model of learning and memory impairment human neuroblastoma cell lines (SH-SY5Y)	↑GSH ↑SOD ↑HO-1 and NQO-1 ↑Nrf2 ↓protein carbonylation level ↓MDA	[72]
Caffeic acid derivative	In vivo	male C57Bl/6 mice model of AD	↓ROS ↑Nrf2 mRNA ↑activation of the Nrf2 signal ↑HO-1 protein	[73]
Gallic acid	In vivo	Drosophila melanogaster model of AD	↓activity of cholinesterases ↓MDA and ROS	[63]
Resveratrol	In vivo	Wistar rats’ model of combined AD and diabetes	↑GSH levels ↑SOD ↓MDA levels	[64]
Resveratrol derivative	In vitro	pheochromocytoma cell line (PC12), mouse microglia BV2 cells	↓NO ↓ROS	[65]
*p*-hydroxybenzyl alcohol	In vitro, In vivo	ICR mice model of AD, human neuroblastoma cell line (SH-SY5Y)	↑Nrf2 protein levels ↑BDNF ↑GDNF	[66]
Taxifolin derivative	In vitro, In vivo	male Swiss mice model of AD, mouse hippocampal nerve cell (HT22)	↑Nrf2 ↑GSH	[67]
Luteolin	In vitro	pheochromocytoma cell line (PC12), rat glioblastoma cell line (C6)	↑Nrf2 ↑NQO1-ARE-response interfering ERK1/2 pathway	[74]
Farrerol	In vitro	mouse microglial BV-2 cells	↑Nrf2/Keap1 pathway ↓ROS and MDA ↑SOD ↑SOD1 and SOD2 mRNA	[75]
Gastrodin	In vitro	hippocampal neurons isolated from Sprague-Dawley rats	↑ SOD ↑mRNA expression of CAT ↑CAT ↑gene expression of Nrf2 ↑ERK1/2 phosphorylation	[76]
Baicalein	In vitro	pheochromocytoma cell line (PC12)	↑Nrf2/HO-1	[77]
Curcuma & garlic-derived hybrids	In vitro	human neuroblastoma cell line (SH-SY5Y)	↑Nrf2	[78]

↑: Increase or up-regulation, ↓: decrease or down-regulation, AChE: acetylcholinesterase, ANT: adenine nucleotide translocator, APE1: apurinic/apyrimidinic endonuclease 1, ARE: antioxidant response element, Aβ: amyloid-beta, BDNF: brain-derived neurotrophic factor, CYP2E1: cytochrome P450 2E1, ERK: extracellular signal-regulated kinases, GDNF: glial cell-derived neurotrophic factor, GFAP: glial fibrillary acidic protein, GSS: glutathione synthetase, HO-1: heme oxygenase-1, IBA1: ionized calcium-binding adaptor molecule 1, IL-1β: interleukin 1 beta, IL-6: interleukin 6, iNOS: inducible nitric oxide synthase, Keap1: kelch-like ECH-associated protein 1, LDH: lactate dehydrogenase, MDA: malondialdehyde, NF-κB: nuclear factor kappa-light-chain-enhancer of activated B cells, NO: nitric Oxide, NQO-1: NAD(P)H quinone dehydrogenase 1, Nrf2: nuclear factor erythroid 2–related factor 2, ROS: reactive oxygen species, SOD: superoxide dismutase, TGF-β: transforming growth factor-beta, TNF-α: tumor necrosis factor-alpha.

**Table 2:** Alkaloids in combating AD through Nrf2/Keap1/ARE and interconnected pathways.

Compounds	Types of Study	Cell Line(s)/Animal Model(s)	Mechanisms of Action	References
Berberine	In vitro	axonal transport impairment induced by calyculin A in wild-type mouse neuroblastoma-2a cell line (N2a)	↑SOD ↓MDA ↓tau and NFs hyperphosphorylation, ↑cell metabolism, ↑cell viability ↑PP-2A activity ↑NF axonal transport	[81]
		glutamate-induced oxidative stress and apoptosis in pheochromocytoma cells (PC12) and neuroblastoma-2a (N2a) cell lines	↓ROS ↓MDA ↑SOD ↑GSH ↓Bax/Bcl-2 ↓caspase-3 ↓DNA fragmentation ↑cell viability	[82]
		homocysteic acid-induced neuronal cell death in murine hippocampal neuronal cell line (HT-22)	↓ROS, ↓LDH, ↓nuclear condensation, ↓necrotic death, ↓cell apoptosis, ↑cell survival, ↑phosphorylated Akt	[83]
	In vivo	LPS-induced learning and memory dysfunctions in the male albino Wistar rats	↑GSH, ↑GPx, ↑SOD, ↑CAT, ↓MDA, ↓3-NT, ↓NF-κB, ↓TLR4, ↓TNF-α, ↓IL-6, ↓COX-2, ↓AChE, ↓DNA fragmentation, ↓caspase-3 ↓protein carbonyl, ↓GFAP, ↑sirtuin 1, ↓p38 MAPK, ↑spatial recognition memory	[84]
		streptozotocin-induced dementia in male Wistar rats	↓ROS, ↑GSH, ↑T-SHs, ↑GST activity, ↓TBARS, ↓protein carbonyl levels, ↑δ-ALA-D, ↑NTPDase, ↑5ʹ-nucleotidase activity, ↑ADA activity, ↑recognition index	[87]
Trigonelline	In vivo	Aβ_1–40_ induced AD in adult male Swiss albino mice	↑SOD, ↑GSH, ↑MMP, ↓MDA, ↓protein carbonyl levels, ↓LDH, ↓COX-2, ↓GFAP, ↓TNF-α, ↓IL-6, ↑spatial recognition memory	[88]
		LPS-induced cognitive impairment in the male albino Wistar rats	↑SOD, ↑GSH, ↓MDA, ↓AChE, ↓TNF-α, ↓IL-6, ↑BDNF, ↑spatial learning, and memory	[89]
Tetramethylpyrazine (Ligustrazine)	In vitro	cobalt chloride-induced neurotoxicity in PC12 cells	↓mitochondrial and intracellular superoxide, ↓ROS, ↑GSH, ↓GSSG, ↑nuclear Nrf2 expression, ↑GCLc expression, ↑Nrf2 transcription activity, ↑ARE-luciferase, ↑cell viability, ↓Bax, ↓cleavage of caspase-3 and -9, ↓PARP, ↑Bcl-2, ↓cytochrome c, ↓cell apoptosis, ↓HIF-1α/NOX2 pathway	[90]
	In vivo	cobalt chloride-induced neurotoxicity in adult male Wistar rats	↑Nrf2, ↓HIF-1α, ↓NOX2 protein expression, ↓cell apoptosis, ↑spatial learning and memory	
Ligustrazine phosphate	In vivo	scopolamine-induced amnesia in male Sprague-Dawley rats	↑SOD, ↑GPx, ↓MDA, ↑behavioral performance	[91]
	In vitro	abdominal skins of male Sprague-Dawley rats	↑penetration ability, ↑drug deposition in skin	
Ligustrazine phosphate and huperzine A	In vivo	scopolamine-induced amnesia in male Sprague-Dawley rats	↑SOD, ↑GPx, ↓MDA, ↑spatial memory	[92]
Huperzine A	In vitro	tert-butyl hydroperoxide-induced oxidative stress in pheochromocytoma cells (PC12) Aβ_25–35_-induced neurotoxicity in PC12 cells	↓ROS, ↑SOD, ↓LDH, ↑cell viability, ↓morphological damage ↑cell viability	[93]
Deoxyvasicine	In vivo	scopolamine-induced cognitive dysfunction in male C57BL/6J mice	↑GPx, ↓TNF-α, ↓AChE, ↑ChAT, ↑BDNF, ↑ACh, ↑spatial learning and memory	[98]
	
HJ22 (a novel derivative of piperine)	In vivo	ibotenic acid-induced cognitive impairment in Sprague-Dawley rats	↓PPI of Keap1-Nrf2, ↑nuclear Nrf2 expression, ↑SOD, ↑CAT activities, ↑GR, ↓MDA, ↑ARE, ↓IL-1β, ↓TXNIP, ↓NLRP3, ↓apoptotic cell death, ↓AChE, ↑ChAT, ↑ACh, ↑Bcl-2/Bax ratio, ↑Nissl body, ↓ASC, ↓caspase-1	[94]
Radical-containing nanoparticles coupled with piperine	In vitro	Aβ_1–42_-induced damage in human neuroblastoma SH-SY5Y cells	↓ROS, ↓hydroxyl radical production, ↑GPx, ↑CAT, ↓MDA, ↓protein carbonyl levels, ↓8-OHdG, ↓DNA fragments, ↑cell viability	[95]
Fangchinoline	In vitro	glutamate-induced oxidative neuronal damage in mouse neuronal cells (HT-22)	↓ROS overproduction, ↑SOD activity, ↑Nrf2 protein level, ↓Keap1 expression, ↑HO-1 protein level, ┴cell morphological damages, ↑cell viability, and regulating Keap1/Nrf-2 antioxidation signaling pathway	[97]
Dauricine	In vitro	Cu^2+^ induced oxidative damage on APPsw cells	↑nuclear Nrf2, ↓Keap1 expression, ↑cell viability, ↓ROS levels, ↑SOD activity, ↑MMP level, ↓Aβ_1–42_ secretion, ↓Bax/Bcl-2 ratio, ↓caspase-3 activity, ↓apoptotic rate	[96]
		Aβ_1–42_-transgenic *Caenorhabditis elegans GMC101*	↓oxidative toxicity of Aβ, ↑survival rates	
Plumbagin	In vivo	streptozotocin-induced AD in adult male Swiss-albino mice	↑activation of Nrf2/ARE pathway, ↓astrogliosis, ↓GFAP expression, ↑spatial learning and memory	[99]
	In silico		↓β-secretase enzyme	
Embelin	In vivo	scopolamine-induced amnesia in Sprague-Dawley rats	↑SOD1, ↑CAT, ↓4-HNE, ↑immature neurons in the SGZ, ↑BDNF expression, ↑CREB1, ↑ACh, ↓Glu, ↓Dopamine, ↓NE, ↑recognition index, ↑memory retention,	[100]
Harmaline	In vivo	scopolamine-induced memory impairments in male C57BL/6 mice	↑SOD, ↑GPx, ↓MDA level, ↓MPO, ↓NO, ↓TNF-α, ↓AChE activity, ↑ChAT activity, ↑ACh, ↑L-Trp, ↑5-HT, ↑L-Glu, ↓γ-GABA, ↑spatial learning and memory,↓MDA level, ↓TNF-α, ↑ChAT activity, ↑ACh, ↑L-Trp,	[102]
Isorhynchophylline	In vitro	Aβ_25–35_-induced neurotoxicity cells (PC12)	↑cell viability, ↓ROS levels, ↑GSH, ↓MDA levels, ↑MMP level, ↓DNA fragmentation, ↓caspase-3 activity, ↑Bcl-2/Bax ratio	[103]
Aloperine	In vitro	neuroblastoma N2a cells co-transfected with Swedish mutant APP and ΔE9 deleted presenilin-1 (N2a/Swe.D9) H_2_O_2_-induced secondary insults in N2a/Swe.D9 cells	↑intracellular GSH levels, ↑GPx activity, ↓generation of ROS, ↓4-HNE, ↑MMP level, ↑intracellular ATP level ↑cell viability, ↓apoptosis, ┴LDH release, ↓translocation of cytochrome c, ↓Bax/Bcl-2 ratio, ↓caspase-3 activity, ┴p38-JNK pathway	[104]
Matrine	In vivo	scopolamine-induced amnesia in male ICR mice	↑T-AOC, ↑SOD, ↑CAT, ↓MDA, ↓AChE activity, ↓BuChE activity, improve learning and memory	[105]
Methyl jasmonate	In vivo	scopolamine-induced cognitive impairment in male Swiss mice	↑SOD, ↑CAT, ↑GSH, ↓MDA, ↓AChE activity, ↑spatial working memory, ↑recognition memory, ↑alternation behaviors,	[106]
Neferine	In vivo	aluminium chloride-induced AD in Wistar rats	↓ROS formation, ↑SOD, ↑CAT, ↑GSH, ↓MDA, ↓LDH, ↓NO, ↓AChE activity, ↓Na^+^K^+^ATPase activity, ↓TNF-α, ↓IL-1β, ↓IL-6, ↓iNOS, ↓COX-2, ↓NF-κB, ↑IKBα, ↑memory and learning ability	[107]
Norcepharadione B	In vitro	hydrogen peroxide (H_2_O_2_)-induced neuronal injury in HT-22 mouse neuronal cells	↑SOD, ↑GSH, ↓MDA, ↓LDH activity, ↑HO-1, ↑Bcl-2/Bax ratio, ↓VSOR Cl^−^ currents, ↓cell apoptosis, ↓cell volume change, ↑phosphorylated Akt	[108]
Vincamine	In vitro	Aβ_25–35_ induced cytotoxicity in PC12 cells	↓ROS levels, ↑SOD, ↑GSH, ↓MDA, ↑Bcl-2/Bax ratio, ↑phospho-Akt/Akt ratio, ↑cell viability, ↓cell apoptosis	[109]
*Corydalis edulis* total alkaloids	In vivo	d-gal induced AD in Sprague-Dawley male rat	↓ROS, ↑SOD, ↑CAT, ↓MDA, ↓TNF-α, ↓IL-1β, ↓Aβ accumulation, ↓NF-κBp65 expression, ↑MAP2, ↑memory and learning ability	[110]
girinimbine, mahanimbine and murrayanine	In vivo	Ageing-induced oxidative stress in male Swiss albino mice	↑GPx, ↑GSH, ↑GRD, ↑SOD, ↑CAT, ↓LPO level, ↓NO levels, ↑ACh, ↓AChE activity	[111]

↑: Increase or up-regulation, ↓: decrease or down-regulation, ┴: blockade or suppressed, Aβ: Amyloid beta, ACh: acetylcholine chloride, AChE: acetylcholinesterase, AD: Alzheimer’s disease, ADA: adenosine deaminase, APPsw: Swedish mutant form of human β-amyloid precursor protein, ARE: antioxidant response element, ATP: adenosine triphosphate, BDNF: brain-derived neurotrophic factor, BuChE: butyrylcholinesterase, CAT: catalase, Ch: choline chloride, ChAT: choline acetyltransferase, COX-2: cyclooxygenase 2, d-gal: d-galactose, GCLc: γ-glutamylcysteine ligase, GFAP: glial fibrillary acidic protein, GPx: glutathione peroxidases, GRD or GR: glutathione reductase, GSH: glutathione, GSSG: oxidized GSH, GST: glutathione *S*-transferase, γ-GABA: γ-aminobutyric acid, HIF-1α: hypoxia-inducible factor 1α, HO-1: heme oxygenase-1, IKBα: NF-κB inhibitor, IL: Interleukin, iNOS: inducible nitric oxide, Keap1: Kelch-like ECH-associated protein 1, LDH: lactate dehydrogenase, LPO: lipid peroxidation, LPS: lipopolysaccharide, l-Glu: l-glutamic acid, l-Trp: l-tryptophan, MAPK: mitogen-activated protein kinase, MAP2: microtubule-associated protein 2, MDA: malondialdehyde, MMP: mitochondrial membrane potential, MPO: myeloperoxidase, NE: Norepinephrine, NFs: neurofilaments, NF-κB: nuclear factorkappa B, NLRP3: nod-like receptor protein 3, NO: nitric oxide, NOX2: nicotinamide oxidase 2, Nrf2: nuclear factor erythroid 2-related factor 2, NTPDase: ecto-nucleoside triphosphate diphosphohydrolase, PARP: Poly (ADP-ribose) polymerase, PPI: protein-protein interaction, PP-2A: Protein phosphatase 2A, ROS: reactive oxygen species, SGZ: subgranular zone, SOD: superoxide dismutase, TBARS: thiobarbituric acid reactive substance, TLR4: toll-like receptor 4, TNF-α: tumor necrosis factor α, T-SHs: total thiols, δ-ALA-D: δ-Aminolevulinic acid dehydratase activity, TXNIP: thioredoxin-interacting protein, T-AOC: total antioxidant capacity, VSOR: volume-sensitive outwardly rectifying, 3-NT: 3-nitrotyrosine, 4-HNE: 4-hydroxy-2-nonenal, 5-HT: 5-hydroxy- tryptamine, 5-HIAA: 5-hydroxyindole-3-acetic acid, 8-OHdG: 8-hydroxy-2ʹ-deoxyguanosine.

**Table 3:** Terpenes and terpenoids in combating AD through Nrf2/keap1/ARE and interconnected pathways.

Compounds	Classification	Types of Study	Cell Line(s)/Animal Model(s)	Mechanisms of Action	References
Carvacrol	monoterpene	In vivo, In vitro	male C57BL/6 mice, hippocampal neurons isolated from neonatal C57BL/6 mice	↑GPx ↑CAT ↑SOD ↓MDA ↑GSH ↓ROS	[113]
α-Pinene				↓ROS	[114]
1,8-Cineole	monoterpene	In vitro	pheochromocytoma cell line (PC12)	↑CAT ↑SOD ↑GPx ↑GR ↑HO-1	
*p*-Cymene	monoterpene	In vivo	male Swiss mice	↓nitrite ↓lipid peroxidation ↑CAT ↑SOD	[115]
Linalool	monoterpenoid	In vivo	male C57BL/6 J mice	↑GPx ↑SOD ↑Nrf2/HO-1	[116]
Thymol				↑Nrf2/HO-1 signaling	[117]
Carvacryl Acetate	monoterpenoid	In vivo, In vitro	male Swiss albino mice, hippocampal neurons isolated from Swiss albino mice	↓lipid peroxidation ↓nitrite contents ↓hydroxyl radical contents ↑GSH ↑CAT ↑GPx ↑ SOD	[118]
Borneol	monoterpenoid	In vitro	human neuroblastoma cell line (SH-SY5Y)	↓ROS ↑Nrf2 ↑HO-1	[119]
Geraniol				↑GSH ↓ROS	[120]
ECN	sesquiterpenoid	In vitro, In vivo	pheochromocytoma cell line (PC12) Male ICR mice	↑Nrf2 ↑HO-1 ↑Nrf2/ARE signaling	[121]
		In vitro	murine microglial cell line (BV-2)	↓ROS production	[122]
Lactucopicrin	sesquiterpenoid	In vitro	neuroblastoma cell lines (N2a), Rat glioblastoma cell line (C6)	↑Nrf2 ↑nerve growth factor ↓ROS ↑mAChR, *p*-Akt, and Bcl-2	[123]
α-Cyperone	sesquiterpenoid	In vitro	murine microglial cell line (BV-2), human neuroblastoma cell line (SH-SY5Y), mouse hippocampal nerve cells (HT22)	↑Akt/Nrf2/HO-1 ↑nuclear tanslocation of Nrf2	[124]
Artemether	sesquiterpenoid	In vitro, In vivo	homozygous 3xTg-AD mouse (34,830-JAX) model of AD, human neuroblastoma cell line (SH-SY5Y), pheochromocytoma cell line (PC12)	↑HO-1 ↑SOD ↑Nrf2 ↓MDA	[125]
Bakkenolide B	sesquiterpenes	In vitro	LPS-induced neuroinflammation in mouse BV2 microglial cells	↑Nrf2, ↑HO-1, ↑NQO1, ↓ROS production, ↑AMPK phosphorylation, ↓IL-1β, ↓IL-6, ↓IL-12, ↓TNF-α, ↓NO, ↓iNOS, ↑cell viability	[126]
Carnosic acid	diterpene	In vitro	human neuroblastoma cell line (SH-SY5Y)	↑PI3K/Akt ↑Nrf2	[127]
		In vitro	rat pheochromocytoma subclone cell line (PC12h)	↑Nrf2	[128]
		In vitro, In vivo	male C57BL/6 mice, Rat pheochromocytoma subclone cell line (PC12h)	↑Nrf2/ARE pathway	[129]
		In vitro	human neuroblastoma cell line (SH-SY5Y)	inducing the metalloprotease gene TACE/ADAM17	[130]
Andrographolide	diterpenoid	In vitro	mouse hippocampal nerve cells (HT22)	↑Nrf2/ARE/HO-1 pathway	[131]
			pheochromocytoma cell line (PC12)	↑Nrf2-mediated p62 signaling pathway	[132]
Compound K	triterpenoid	In vitro, In vivo	male C57BL/6 mice, mouse hippocampal nerve cells (HT22)	↑Nrf2 ↑HO-1 ↑quinone oxidoreductase 1	[133]
Ginsenoside	triterpenoi	In vitro	human neuroblastoma cell line (SH-SY5Y)	↑activation Nrf2	[134]
Gedunin	triterpenoid	In vitro	immortalized microglial cell line (IMG), human neuroblastoma cell line (SH-SY5Y)	↓NO ↓NF-κB ↑Nrf2	[135]
Lycopene	tetraterpene	In vitro, In vivo	male C57BL/6J mice, murine microglial cell line (BV-2)	↑Nrf2 ↑HO-1 ↑NQO-1	[136]
Gypenoside xvii	tetraterpenoid	In vitro	pheochromocytoma cell line (PC12)	↑Nrf2/ARE/HO-1 pathways	[137]

↑: Increase or up-regulation, ↓: decrease or down-regulation, ADAM17: ADAM metallopeptidase domain 17, Akt: protein kinase B (PKB), ARE: antioxidant response element, Bcl-2: B-cell lymphoma 2, ECN: 7β-(3-ethylcis-crotonoyloxy)-1α-(2-methylbutyryloxy)3,14-dehydro-*Z*-notonipetranone, HO-1: heme oxygenase-1, Keap1: kelch-like ECH-associated protein 1, mAChR: muscarinic acetylcholine receptor, NQO-1: NAD(P)H quinone dehydrogenase 1, Nrf2: nuclear factor erythroid 2-related factor 2, *p*-Akt: phospho-protein kinase B, PI3K: phosphoinositide 3-kinases, ROS: reactive oxygen species, SOD: superoxide dismutase.

**Table 4:** Carotenoids combating AD through Nrf2/Keap1/ARE and interconnected pathways.

Compounds	Types of Study	Cell Line(s)/Animal Model(s)	Mechanisms of Action	References
Astaxanthin	In vitro	glutamate-induced neurotoxicity in mouse neuronal cell line (HT-22)	↓intracellular ROS accumulation, ↑ARE, ↑nuclear Nrf2, ↑HO-1, ↑Bcl-2/Bax ratio, ↓PARP, ↓caspase-3/8/9 activity, ↓cytochrome c, ↓LDH, ↓AIF, ↑*p*-Akt, ↑*p*-GSK-3β (Ser9), ↑cell viability	[144]
	In vivo	scopolamine-induced spatial learning deficits in *Swiss albino* male mice	↑SOD, ↑CAT, ↓NO, ↑spatial learning and memory	[145]
	In vivo	Aβ_1–42_-induced AD in adult male Wistar rats	↑GPx, ↓MDA, ↓superoxide anion, ↓protein carbonyl levels, ↓neuronal degeneration, ↓positive staining of Aβ, ↑spatial learning and memory	[146]
	In vivo	LPS-induced mice AD model	↓ROS, ↑GSH, ↑GSH/GSSG ratio, ↓thiobarbituric acid, ↓NO, ↓β-secretase activity, ↓APP level, ↓BACE1, ↓Aβ_1–42_, ↓COX-2, ↓GFAP, ↓IBA-1, ↓iNOS, ↓TNF-α, ↓IL-1β, ↓IL-6, ↓MCP-1, ↓MIP-1α, ↓MIP-1β, ↓STAT3, ↑spatial learning and memory	[147]
	In vitro	BV-2 microglial cells	↓NO, ↓TBARS, ↓β-secretase, ↓APP level, ↓BACE1, ↓COX-2, ↓IBA-1, ↓iNOS, ↓TNF-α, ↓IL-1β, ↓IL-6, ↓MCP-1, ↓MIP-1α, ↓MIP-1β, ↓STAT3	
	In vitro	*tert*-butyl hydroperoxide- induced oxidative stress in pheochromocytoma cell line (PC12)	↓ROS, ↑SOD, ↓MDA, ↓LDH release, ↑cell viability, ↓morphological damage	[93]
		Aβ_25–35_-induced neurotoxicity in PC12 cells	↑cell viability	
Astaxanthin and Huperzine A	In vitro	*tert*-butyl hydroperoxide- induced oxidative stress in PC12 cells Aβ_25–35_-induced neurotoxicity in PC12 cells	↓ROS, ↑SOD, ↓MDA, ↓LDH, ↑cell viability, ↓morphological damage ↑cell viability	[93]
Crocin	In vitro	L-glutamate-damaged HT-22 mouse neuronal cells	↓intracellular ROS, ↓MMP dissipation, ↓overload of Ca^2+^, ↑Bcl-xL, ↓Bax, ↓Bad, ↓cleaved caspase-3, ↓apoptosis rate, ↑cell viability, ↑phosphorylation of Akt and mTOR	[150]
	In vivo	AlCl_3_/d-gal-induced AD in BALB/c mice	↓ROS, ↑GPx, ↑SOD, ↓Aβ_1–42_ deposition, ↓AChE, ↑ChAT, ↑ACh, ↑memory abilities and cognitive functions	
	In vivo	malathion-induced spatial memory deficits in adult male Wistar rats	↑GSH, ↓MDA, ↓TNF-α, ↓IL-6, ↓tau hyperphosphorylation, ↑PSD93 protein level, ↓caspase-3/8/9 activity, ↓Bax/Bcl-2 ratio, ↓cell apoptosis, ↑spatial learning and memory	[152]
		streptozotocin-induced spatial memory deficit and oxidative stress in adult male Wistar Albino rats	↑GPx activity, ↑total thiol concentration, ↓MDA	[153]
				[154]
Lycopene	In vitro	LPS-treated BV2 microglial cells	↓intracellular ROS generation, ↑MMP, ↑Nrf2, ↑HO-1 expression, ↑NQO-1 expression, ↓*p*-ERK, ↓*p*-JNK, ↓*p*-p38, ↓*p*-AKT, ↓NF-κB nuclear translocation, ↓*p*-IκB, ↑Nrf2	[136]
	In vivo	LPS-induced learning and memory loss in male C57BL/6J mice	↑GSH, ↑SOD, ↑CAT, ↓Aβ_1–42_ accumulation, ↓APP level, ↓BACE1 expression, ↑ADAM10, ↓IBA-1, ↓COX-2, ↓iNOS, ↓IL-1β, ↑IL-10, ↓MMP-9 expression, ↑spatial learning and memory	
	In vivo	Tau transgenic mice expressing P301L mutation	↑GPx activities, ↓MDA levels, ↓tau hyperphosphorylation, ↑spatial and passive memory	[156]
Strigolactone analogue (GR24rac)	In vitro	LPS-treated SIM-A9 mouse microglial cells	↑Nrf2 nuclear level, ↑HO-1, ↑NQO-1, ↓NO, ↓iNOS, ↓TNF-α, ↓IL-1β, ↓COX-2, ↓NF-κB, ↑PPARγ expression	[160]
		LPS-treated BBB bEnd.3 mouse brain endothelial cells	↓TNF-α, ↓IL-1β, ↑NQO-1	
β-carotene	In vivo	streptozotocin-induced AD in adult male Swiss albino mice	↑GSH, ↑SOD, ↑CAT, ↓GSSG/GSH ratio, ↓AChE activity, ↓Aβ_1–40_ and Aβ_1–42_ levels, ↑cognitive performance	[157]
	In silico		↓AChE activity	
Levocarnitine	In vivo	AlCl_3_-induced spatial working memory deficits in adult male Swiss albino mice	↓GSH, ↓MDA, ↓NO, ↓AOPP levels, ↑spatial working memory performance	[158]

↑: Increase or up-regulation, ↓: decrease or down-regulation, ┴: blockade or suppressed, Aβ: Amyloid beta, ACh: acetylcholine chloride, AChE: acetylcholinesterase, AD: Alzheimer’s disease, ADAM 10: a disintegrin and metalloprotease 10, AIF: apoptosis-inducing factor, Akt: protein kinase B, AlC13: aluminum trichloride, AOPP: advanced oxidation of protein products, APP: amyloid precursor protein, ARE: antioxidant response element, BACE1: β-secretase 1, BBB: blood–brain barrier, CAT: catalase, ChAT: choline acetyltransferase, COX-2: cyclooxygenase 2, d-gal: d-galactose, ERK: extracellular signal-regulated kinases, GFAP: glial fibrillary acidic protein, GPx: glutathione peroxidases, GSH: glutathione, GSK-3β: glycogen synthase kinase 3 beta, GSSG: oxidized GSH, HO-1: heme oxygenase-1, IBA-1: ionized calcium binding adaptor molecule 1, IL: Interleukin, iNOS: inducible nitric oxide, JNK: C-Jun N-terminal Kinase, LDH: lactate dehydrogenase, LPS: lipopolysaccharide, MDA: malondialdehyde, MMP: mitochondrial membrane potential, MMP-9: matrix metallopeptidase 9, mTOR: mammalian target of rapamycin, NF-κB: nuclear factorkappa B, NO: nitric oxide, Nrf2: nuclear factor erythroid 2-related factor 2, NQO-1: NAD(P)H dehydrogenase [quinone] 1, PARP: Poly (ADP-ribose) polymerase, PPARγ: peroxisome proliferator-activated receptor γ, PSD93: postsynaptic density protein 93, ROS: reactive oxygen species, SOD: superoxide dismutase, STAT3: Signal transducer and activator of transcription 3, TNF-α: tumor necrosis factor α, TBARS: Thiobarbituric acid reactive substance.

**Table 5:** Sulfur compounds in combating AD through Nrf2/Keap1/ARE and interconnected pathways.

Compounds	Types of Study	Cell Line(s)/Animal Model(s)	Mechanisms of Action	References
Sulforaphane	In vitro	mouse neuroblastoma cell line (N2a)	↓ROS ↓MDA ↑SOD ↑Nrf2	[164]
		human neuroblastoma cell line (SH-SY5Y)	↑GSH ↑GR ↑glutathione transferase	[165]
		hippocampal neuron isolated from C57Bl6J mice	↑activation of Nrf2/ARE pathway	[166]
		neuronal cell isolated from the Wistar rat’s striatum	↑HO-1 ↑GSH ↑Nrf2/ARE pathway	[167]
		pheochromocytoma cell line (PC12)	↑HO-1 ↑translocation of Nrf2 ↑PI3K/Akt	[168]
	In vivo	male C57Bl/6 mice model of PD	↑GSH ↑GST ↑GR	[169]
	In vitro, In vivo	male C57Bl/6 mice model of PD, human neuroblastoma cell line (SH-SY5Y)	↓ROS ↓MDA ↑GSH ↑Nrf2 ↑HO-1	[170]
	In vitro	human microglia-like THP-1 cells	↑Nrf2/HO-1	[171]
		astrocyte isolated from (P1eP2) Sprague-Dawley rats		[173]
		pheochromocytoma cell line (PC12)		[174]
*S*-allyl cysteine	In vivo	Swiss albino mice model of experimental dementia of Alzheimer’s type	↑GSH ↑GPx	[175]
	In vitro, In vivo	Nrf2 heterozygous mice, Nrf2 knockout mice, neuronal cell isolated from the Sprague-Dawley rat embryos	↑Nrf2-dependent antioxidant responses	[176]
	In vivo	male C57BL/6 mice	↑Nrf2 transcription factor	[177]
	In vitro	pheochromocytoma cell line (PC12)	↓apoptosis	[178]
	In vitro	pheochromocytoma cell line (PC12)	↑mitochondrial membrane potential	[179]
	In vitro	hippocampal and cerebellar granule neurons isolated from embryos of Wistar rats	↓ROS	[180]
S-ethyl cysteine, S-propyl cysteine	In vivo	male C57BL/6 mice	↓MDA ↑GSH ↓ROS ↑GPx ↑SOD ↑CAT	[181]


6-(Methylsulfinyl) hexyl isothiocyanate	In vitro	neuronal cell isolated from the Wistar rat’s striatum	↑HO-1 ↑GSH ↑Nrf2/ARE pathway	[167]
	In vivo	male C57Bl/6 mice model of AD	↓ROS ↑GSH ↑Nrf2/ARE pathway	[182]
Allicin	In vitro	human RPE cell line (ARPE-19)	↓ROS ↓MDA ↑GSH/glutathione disulfide ratio	[183]
	In vivo	male C57BL/6 mice	↑Nrf2/ARE ↑GSH levels ↓ROS levels ↑GPx	[184]
	In vivo	male Sprague-Dawley rats	↑PERK and Nrf2 ↓ROS levels ↑GSH level ↓lipid peroxidation	[185]
Thiacremonone	In vitro, In vivo	APP/PS1 transgenic mice model, neuronal cells isolated from the Sprague–Dawley (SD) rats	↑GSH ↓NF-κB	[186]
3*H*-1,2-Dithiole-3- thione	In vivo	Tg2576 AD mouse model	↑Nrf2 ↑HO-1 ↑Sirt1/Nrf2	[187]
Hydrogen sulfide	In vitro	mouse hippocampal nerve cell line (HT22)	↑GSH ↑cysteine ↑K_ATP_ channels ↑Cl^−^ channels	[188]
Lipoic acid	In vitro, In vivo	female C57BL/6 mice, retinal neuronal cell line (RGC-5)	↑Nrf2 ↑HO-1 ↑Keap1/Nrf2 ↓ROS	[189]

↑: Increase or up-regulation, ↓: decrease or down-regulation, Akt: protein kinase B (PKB), ARE: antioxidant response element, HO-1: heme oxygenase-1, Keap1: kelch-like ECH-associated protein 1, MDA: malondialdehyde, NF-κB: nuclear factor kappa-light-chain-enhancer of activated B, Nrf2: nuclear factor erythroid 2-related factor 2, PERK: PKR-like endoplasmic reticulum (ER) kinase, PI3K: phosphoinositide 3-kinases, ROS: reactive oxygen species, SOD: superoxide dismutase, TACE: tumor necrosis factor-α-converting enzyme.

**Table 6:** Miscellaneous compounds in combating AD through Nrf2/Keap1/ARE and interconnected pathways.

Compounds	Classification	Types of Study	Cell Line(s)/Animal Model(s)	Mechanisms of Action	Reference
Compound K	ginsenoside	In vivo	scopolamine hydrobromide-induced memory impaired in ICR mice	↑Nrf2, ↓Keap1, ↑HO-1, ↑SOD, ↑GPx, ↓MDA, ↓Aβ expression, ↓neuronal apoptosis, ↓Bax, ↑Bcl-2, ↓caspase-3 activity, ↓APP expression, ↓BACE1, ↓PS1 expression, ↑spatial cognition and memory function, normalize neuronal morphology	[190]
		In vitro	glutamate-induced cytotoxicity in mouse hippocampal cells (HT22),	↑Nrf2, ↑HO-1, ↑NQO1, ↑GR, ↓apoptotic cells	[133]
		In vivo	scopolamine-induced memory impaired in male C57BL/6 mice	↑Nrf2-mediated antioxidant enzyme, ↑spatial learning and memory	
20(*S*)-Protopanaxadiol	ginsenoside	In vivo	scopolamine-induced memory deficit in ICR male mice	↑SOD, ↓MDA, ↓AChE, ↑ACh, ↑spatial learning and memory	[191]
Ginsenoside Rd	ginsenoside	In vitro	Aβ_25–35_-induced neuronal damage in primary cultured hippocampal neurons	↓ROS, ↑SOD, ↑GPx, ↓MDA, ↓loss of hippocampal neurons, ↓cytochrome c, ↓Bax, ↑Bcl-2, ↑neuronal survival	[193]
20(*S*)-Protopanaxatriol	ginsenoside	In vivo	scopolamine-induced cognitive deficits in male mice	↑SOD, ↓MDA, ↓AChE, ↑ACh content, ↑ChAT activity, ↑spatial memory	[194]
Ginsenoside Rg1	ginsenoside	In vivo	chronic restraint stress-induced learning and memory impairments in adult male Kunming mice	↓ROS, ↑SOD, ↓MDA, ↓8-OHdG, ↓NOX2 expression, ↑learning and memory function	[195]
Pseudoginsenoside- F11	saponin	In vivo	Tg-APPswe/PS1dE9 (APP/PS1) mice,	↑SOD, ↑GPx, ↓MDA, ↓APP, ↓Aβ_1–40_, ↓caspase-3 activity, ↓JNK 2, ↓p53, ↓learning and memory impairment	[197]
			Aβ_1–42_-induced AD in male Kunming mice	↑spatial learning and memory	
Timosaponin B-II	saponin	In vivo	scopolamine-induced AD in male Kunming mice	↑SOD, ↑GPx, ↓MDA, ↓AChE, ↑spatial learning and memory	[198]
Aloe-emodin	anthraquinone	In vitro	hydrogen peroxide (H_2_O_2_)-induced cytotoxicity in PC12 cells,	↓intracellular ROS accumulation, ↓NO, ↓LDH, ↑cell viability	[199]
		In vivo	scopolamine-induced memory impairment in Kunming mice	↑SOD, ↑GPx, ↓MDA, ↓AChE, ↑ACh content, ↑spatial learning and memory	
Methysticin	kavalacton	In vivo	52-weeks old transgenic APP/Psen1 mice	↑HO-1, ↑GCLC expression, ↑Nrf2/ARE pathway, ↓microglia activation, ↓astrogliosis, ↓GFAP, ↓IBA-1, ↓TNF-α, ↓IL-17A, ↓memory loss	[200]
α-Tocopherol	vitamins	In vitro	Aβ_1–42_-induced neurotoxicity in SH-SY5Y neuroblastoma cells	↑Nrf2, ↓iNOS, ↓APP processing, ↑cell viability	[201]
α-Tocopherol quinine	vitamins	In vivo	Memory impairment in APPswe/PS1dE9 transgenic mice (transgenic mice with AD)	↓ROS, ↑SOD, ↓MDA, ↓NF-κB, ↓IBA-1 protein levels, ↓iNOS, ↓IL-1β, ↓IL-6, ↓Aβ oligomer levels, ↓microglia activation, ↑spatial cognitive performance	[202]
		In vitro	microglial cells (BV-2)	↓NF-κB, ↓IBA-1	
α-Linolenic acid	fatty acid	In vitro	Aβ_25–35_-induced neurotoxicity in C6 glial cells	↑Nrf2, ↑HO-1, ↓ROS, ↑neprilysin, ↑IDE expression, ↓NO, ↓TNF-α, ↓IL-6, ↓iNOS, ↓COX-2, ↓Aβ accumulation, ↑cell viability	[203]
Chitosan	polysaccharide	In vitro	H_2_O_2_/FeSO_4_- induced cell death in the NT2 neural cells	↑Nrf2, ↑HO-1, ↑GSH, ↑γ-GCS, ↑Hsp-70, ↓NF-κB, ↓caspase-3, ↓Aβ formation, ↑cell viability	[211]
Shikonin	naphthoquinone	In vitro	Aβ_1–42_-induced neurotoxicity in PC12 cells	↓ROS, ↑SOD, ↑GPx, ↑CAT, ↓MDA, ↑MMP, ↓LDH, ↓caspase-3, ↑Bcl-2/Bax ratio, ↑cell viability	[207]
*Lycium barbarum* polysaccharide	polysaccharide	In vitro	H_2_O_2_- induced neurotoxicity in PC12 cell	↑Nrf2/HO-1, ↑ARE-luciferase activity, ↓ROS, ↓mitochondrial apoptosis, ↓caspase-3 and -9 activity, ↑cell viability	[212]
		In vivo	CoCl_2_-induced neurotoxicity in male Wistar rats	↑Nrf2/HO-1 expression, ↓apoptosis, ↑spatial learning and memory abilities	
*Amanita caesarea* polysaccharides	polysaccharide	In vitro	glutamate-induced cytotoxicity in HT22 mouse hippocampal neuronal cells,	↓intracellular ROS accumulation, ↑Nrf2, ↓Keap1, ↑HO-1, ↑GCLC expression, ↓cytochrome c, ↑MMP, ↓Bax, ↑Bcl-2, ↓caspase-3, ↑cell viability, ↓apoptotic rate,	[213]
		In vivo	AlCl_3_/d-gal-induced AD in BALB/c male mice	↓ROS, ↑SOD, ↑GPx content, ↓Aβ_1–42_ deposition, ↓AChE, ↑ACh content, ↑ChAT activity, ↓memory impairment	
*Inonotus obliquus* polysaccharides	polysaccharide	In vitro	l-glutamic acid-induced cytotoxicity in HT22 mouse hippocampal neuronal cells,	↑Nrf2, ↓Keap1, ↑HO-1, ↑SOD-1, ↑GCLC, ↓intracellular ROS accumulation, ↓LDH, ↑MMP, ↓Bax, ↑Bcl-2, ↓caspase-3 activity, ↑cell viability, ↓apoptotic rate	[214]
		In vivo	APP/PS1 transgenic male mice	↓ROS, ↑SOD, ↑GPx content, ↓MDA, ↑Nrf2, ↓Keap1, ↑HO-1, ↑SOD-1, ↑GCLC levels, ↓Aβ_1–42_ deposition, ↓neuronal fiber tangles deposition, ↓4-HNE, ↑memory and cognition function	
Schisanhenol	tannin	In vivo	scopolamine-induced cognitive impairment in male Kunming mice	↑SOD, ↑GPx, ↓MDA, ↓AChE activity, ↓phosphorylated Tau protein, ↑Sirtuin 1 expression, ↑PGC-1α, ↑learning and memorial ability	[215]

↑: Increase or up-regulation, ↓: decrease or down-regulation, Aβ: Amyloid beta, ACh: acetylcholine chloride, AChE: acetylcholinesterase, AD: Alzheimer’s disease, AMPK: AMP-activated protein kinase, APP: Amyloid precursor protein, ARE: antioxidant response element, BACE1: β-secretase 1, CAT: catalase, COX-2: cyclooxygenase-2, ChAT: choline acetyltransferase, DPPH: 1,1-Diphenyl-2-picrylhydrazyl, GCLc: γ-glutamylcysteine ligase, GFAP: glial fibrillary acidic protein, Glc: glucose, GPx: glutathione peroxidases, GSH: glutathione, GR: glutathione reductase, GSK-3β: Glycogen synthase kinase 3 beta, γ-GCS: γ-glutamylcysteine synthetase, HO-1: heme oxygenase-1, HSP70: Heat shock protein-70, IBA-1: ionized calcium binding adaptor molecule 1, IDE: insulin-degrading enzyme, IL: Interleukin, iNOS: inducible nitric oxide, JNK 2: c-Jun N-terminal kinase 2, Keap1: Kelch-like ECH-associated protein 1, LDH: lactate dehydrogenase, LPO: lipid peroxidation, LPS: lipopolysaccharide, MDA: malondialdehyde, MitoSOX: mitochondrial superoxide, MMP: mitochondrial membrane potential, NF-κB: nuclear factorkappa B, NO: nitric oxide, NOX2: NADPH oxidase 2, Nrf2: nuclear factor erythroid 2-related factor 2, NQO-1: NAD(P)H dehydrogenase [quinone] 1, PARP: Poly (ADP-ribose) polymerase, PGC-1α: PPARγ coactivator 1-α, PS1: presenilin 1, ROS: reactive oxygen species, SOD: superoxide dismutase, TNF-α: tumor necrosis factor α, X/XO: xanthine/xanthine oxidase, 4-HNE: 4-Hydroxy-2-Nonenal, 8-OHdG: 8-hydroxy-2ʹ-deoxyguanosine.
